# Plasmonic-Assisted Thermocyclizations in Living Cells Using Metal–Organic Framework Based Nanoreactors

**DOI:** 10.1021/acsnano.1c07983

**Published:** 2021-10-18

**Authors:** Carolina Carrillo-Carrión, Raquel Martínez, Ester Polo, María Tomás-Gamasa, Paolo Destito, Manuel Ceballos, Beatriz Pelaz, Fernando López, José L. Mascareñas, Pablo del Pino

**Affiliations:** † Centro Singular de Investigación en Química Biolóxica e Materiais Moleculares (CiQUS), Departamento de Física de Partículas, Universidade de Santiago de Compostela, 15782 Santiago de Compostela, Spain; ‡ Centro Singular de Investigación en Química Biolóxica e Materiais Moleculares (CiQUS), Departamento de Bioquímica y Biología Molecular, Universidade de Santiago de Compostela, 15782 Santiago de Compostela, Spain; § Centro Singular de Investigación en Química Biolóxica e Materiais Moleculares (CiQUS), Departamento de Química Orgánica, Universidade de Santiago de Compostela, 15782 Santiago de Compostela, Spain; ∥ Centro Singular de Investigación en Química Biolóxica e Materiais Moleculares (CiQUS), Departamento de Química Inorgánica, Universidade de Santiago de Compostela, 15782 Santiago de Compostela, Spain; ⊥ Misión Biológica de Galicia, Consejo Superior de Investigaciones Científicas (CSIC), 36080 Pontevedra, Spain

**Keywords:** bio-orthogonal chemistry, nanocomposites, thermoplasmonics, MOF, intracellular thermocyclization, thermolabile protecting groups

## Abstract

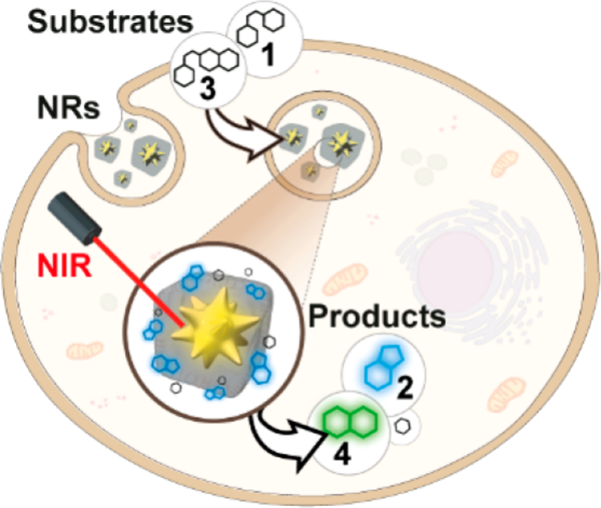

We describe a microporous plasmonic nanoreactor to carry out designed near-infrared (NIR)-driven photothermal cyclizations inside living cells. As a proof of concept, we chose an intramolecular cyclization that is based on the nucleophilic attack of a pyridine onto an electrophilic carbon, a process that requires high activation energies and is typically achieved in bulk solution by heating at ∼90 °C. The core–shell nanoreactor (NR) has been designed to include a gold nanostar core, which is embedded within a metal–organic framework (MOF) based on a polymer-stabilized zeolitic imidazole framework-8 (ZIF-8). Once accumulated inside living cells, the MOF-based cloak of NRs allows an efficient diffusion of reactants into the plasmonic chamber, where they undergo the transformation upon near-IR illumination. The photothermal-driven reaction enables the intracellular generation of cyclic fluorescent products that can be tracked using fluorescence microscopy. The strategy may find different type of applications, such as for the spatio-temporal activation of prodrugs.

Living cells respond to external signals through cascades of connected chemical reactions that take place at physiological temperatures. Most of these reactions are catalyzed by enzymes and have been selected by evolution to ensure an appropriate functioning of living organisms.^1^ A major current goal in chemical and cell biology consists of the discovery of chemically engineered, intracellular reactions that allow to implement non-native functions, thereby influencing the properties of cells in a predictable manner.^2^ The last two decades have witnessed sustained progress in the development of a variety of bio-orthogonal and biocompatible reactions, most of which are based on the use of strained reactants^3−5^ or of metal catalysts.^6−11^ Some of these reactions have already allowed impressive applications either in the activation of prodrugs,^12−15^ or in interrogating biological processes.^16^ However, these approaches are not devoid of important limitations, such as the intrinsic reactivity of the strained reactants, or the problems of biocompatibility and efficiency of the metal-based reagents.^2^

An alternative to these reactions could be based on the development of thermal-driven processes, as this would allow the use of stable reactants and trigger the reactivity using a thermal stimulus; however, it is obvious that cells cannot be heated above physiological temperatures. We envisioned that this problem could be solved by using plasmonic nanoparticles (NPs) as near-IR (NIR) responsive thermal transducers, provided the chemical reaction can be located near the heating source. This requires a straightforward access of the reactants near the NPs, while avoiding the accumulation of “sticky” biomolecules of the biological milieu in the surface of the nanocore.^17^

Herein we demonstrate the viability of this type of photothermal approach by reporting a thermal-promoted chemical reaction, in this case an intramolecular nucleophilic substitution (thermocyclization), that can be performed inside living mammalian cells (Figure 1a). We show that locating gold nanostars (NS from now) inside appropriately designed ZIF-8 NPs provides for very efficient NIR-induced reactions (Figure 1b). Key for the success of the approach is the use of the microporous structure of the ZIF-8 based nanoshell, which allows an efficient internal flow of the small-size reactants near the heating source (NS core) under NIR excitation while blocking access to large biomolecules such proteins. Importantly, the thermal conductivity of ZIF-8 is very low (∼0.1 W/m·K) compared to that of water (0.6 W/m·K) or other common porous coatings such alumina (4–30 W/m·K) or silica (1.4 W/m·K); this property facilitates thermal confinement following irradiation, as it is inversely proportional to the thermal conductivity of the surrounding medium around the heating source.

**Figure 1:**
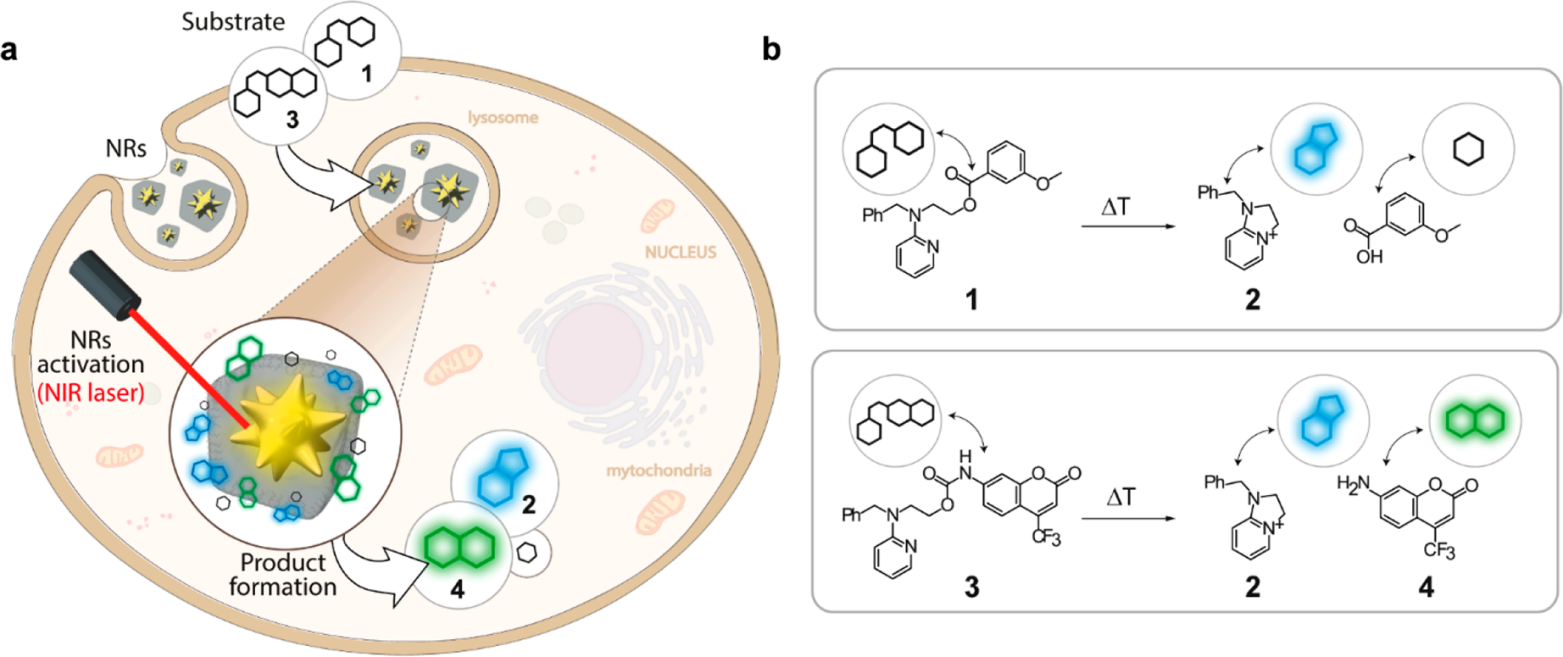
Core–shell MOF-based plasmonic nanoreactor (NR) for intracellular photothermal reactions. (a) NRs featuring a gold nanostar (NS) coated by a porous polymer-modified ZIF-8 cloak are colloidally and structurally stable in aqueous media and work as heterogeneous photo-gated NRs capable of transforming thermolabile substrates inside living cells (thermocyclization). (b) Reacting probes used in this study (substrates **1** and **3**) and fluorescent products (**2** and **4**) resulting from a thermoplasmonic-promoted reaction (Scheme S1 and Figures S1 and S2). Thermocyclization of substrate **1** provides fluorescent product **2** and releases an aromatic carboxylic acid. In contrast, thermocyclization of substrate **3**, in addition to product **2**, generates fluorescent amino-coumarine **4** in a reaction that entails a decarboxylation.

The use of the photothermal properties of plasmonic NPs for photoinduced local heating is well-documented.^18^ These properties are particularly useful for biological applications because the heating can be triggered by NIR light sources,^19^ which excite the corresponding plasmon band of anisotropic metal NPs, usually gold NPs^20,21^ such as nanorods,^22^ nanostars,^23^ and nanoprisms.^24^ The thermoplasmonic approach has also been used in photothermal therapy (analogous to magnetic-induced hyperthermia for treatment of solid tumors) and NIR-gated delivery of payloads in tissues and cells.^18,25^ However, plasmonic photocatalysis, with both hot carrier and thermal contributions, has also been demonstrated for various reactions such as ammonia decomposition,^26^ oxygen dissociation,^27^ and carbon dioxide hydrogenation,^28^ among others.^29,30^ These transformations typically require pulsed illumination, which in an irradiance-dependent manner can lead to NP reshaping and vapor nanobubbles.^31,32^ However, the use of thermoplasmonic heating effects for achieving thermal-promoted chemical reactions in biological and living settings, either with pulsed or continuous wave (CW) light sources, has been rarely reported. A recent report on the intracellular removal of propargyloxycarbonyl groups using silica-plasmonic constructs is based on the action of catalytic gold nanocrystals, in which NIR irradiation seems to accelerate the reaction.^33^ Moreover, it deals with a typical uncaging that might be alternatively performed with palladium catalysts^34^ or with NP-based gold catalysts without light stimulation.^35^

## Results and Discussion

Our initial work in this topic was encouraged by the recent discovery that the modification of ZIF-8 structures with the amphilipic polymer poly[isobutylene-*alt*-maleic anhydride]-*graft*-dodecyl (PMA, Scheme S2) renders the nanocomposites stable in aqueous media and even inside living cells.^23,36^ We have comprehensively characterized the physicochemical properties of inorganic nanoparticles (gold and palladium) equipped with ZIF-8/PMA coatings in a previous work.^23,36^ We hypothesized that placing a plasmonic core (NS) in the interior of the microporous ZIF-8 based shell might produce light-responsive nanoreactors (NRs) that could work in the interior of cells. Other NRs entailing plasmonic NPs immobilized in mesoporous materials such as alumina^27^ and silica have been described, yet always in nonbiological contexts.^37^ More recently, different MOF-based combinations have been proposed for thermoplasmonic catalysis in solar energy conversion.^38^ Moreover, nanocomposites comprising silica-supported plasmonic NPs have been also used for encapsulation of drugs, which can then be intracellularly released upon resonant plasmonic excitation.^39^

**Figure 2:**
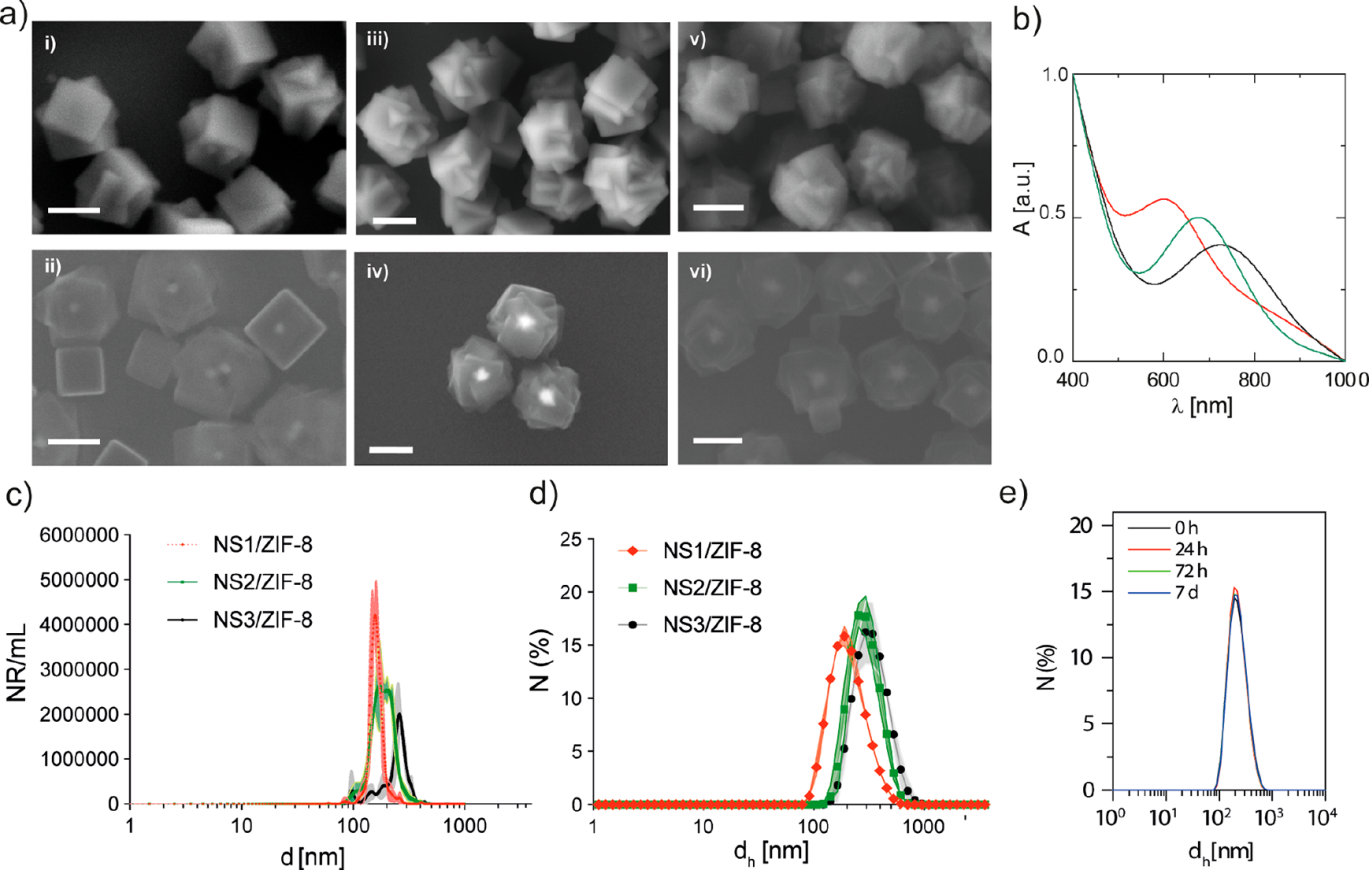
(a) SEM images of the NRs taken at 3 kV (above) and 20 kV (below): NS1/ZIF-8 (i, ii; *d*_NR1_ = 219 ± 25 nm), NS2/ZIF-8 (iii, iv; *d*_NR2_ = 274 ± 30 nm), and NS3/ZIF-8 (v, vi; *d*_NR3_ = 331 ± 34 nm). Scale bars correspond to 200 nm. (b) UV–visible (UV–vis) absorption spectra of NR: NS1/ZIF-8 (red line, SPR band centered at 695 nm), NS2/ZIF-8 (green line, SPR band centered at 770 nm), and NS3/ZIF-8 (black line, SPR band centered at 820 nm). (c) NTA size distribution of NR particles dispersed in water: NSI/ZIF-8 (red line), NS2/ZIF-8 (green line), and NS3/ZIF-8 (black line). (d) DLS number distributions of the hydrodynamic diameter (*d*_h_) of NR particles dispersed in water after their preparation: NSI/ZIF-8 (red line, *d*_h_,_NS1_ = 214 ± 1 nm), NS2/ZIF-8 (green line, *d*_h_,_NS2_ = 301 ± 12 nm), NS3/ZIF-8 (black line, *d*_h_,_NS3_ = 347 ± 59 nm). (e) DLS number distributions of the hydrodynamic diameter (*d*_h_) of NS2/ZIF-8 particles dispersed in water at different time points after their preparation.

Following previously used protocols detailed in the “Materials and Methods” section, we synthesized the desired nearly monodisperse NRs with diameters between 200 and 300 nm as determined by electron microscopy (Figures 2a and S3–S5). The core–shell nanocomposite contains a single plasmonic nanoparticle per NR, which is responsible for the light-associated properties. We made several NRs equipped with plasmonic gold nanoparticles that present different shapes and tunable plasmonic absorption bands: gold nanostars with various sizes and tip-to-tip lengths (NS1, NS2, and NS3, Figure 2b) and gold nanorods (GNRs, Figure S6).

The coating of the NRs with the amphiphilic PMA polymer ensures their colloidal stability, as confirmed by nanoparticle tracking analysis (NTA, Figure 2c and Table S1) and dynamic light scattering measurements (DLS; Figures 2d,e, S7, and S8 and Table S1). NTA measurements allowed us to quantify the number of NRs per mL (Figure 2c). The size and the tip-to-tip distance of the NS core (*d*_NS1_ = 70 ± 10 nm, *d*_NS2_ = 84 ± 10 nm, and *d*_NS3_ = 133 ± 16 nm) determine the final hydrodynamic diameter of the NRs (*d*_h_,_NS1_ ∼ 215 nm, *d*_h,NS2_ ∼ 300 nm, and *d*_h,NS3_ ∼ 350 nm). The PMA-functionalized NRs remain colloidally stable in water and cell medium over time as confirmed by DLS (Figures 2e and S9).

**Figure 3:**
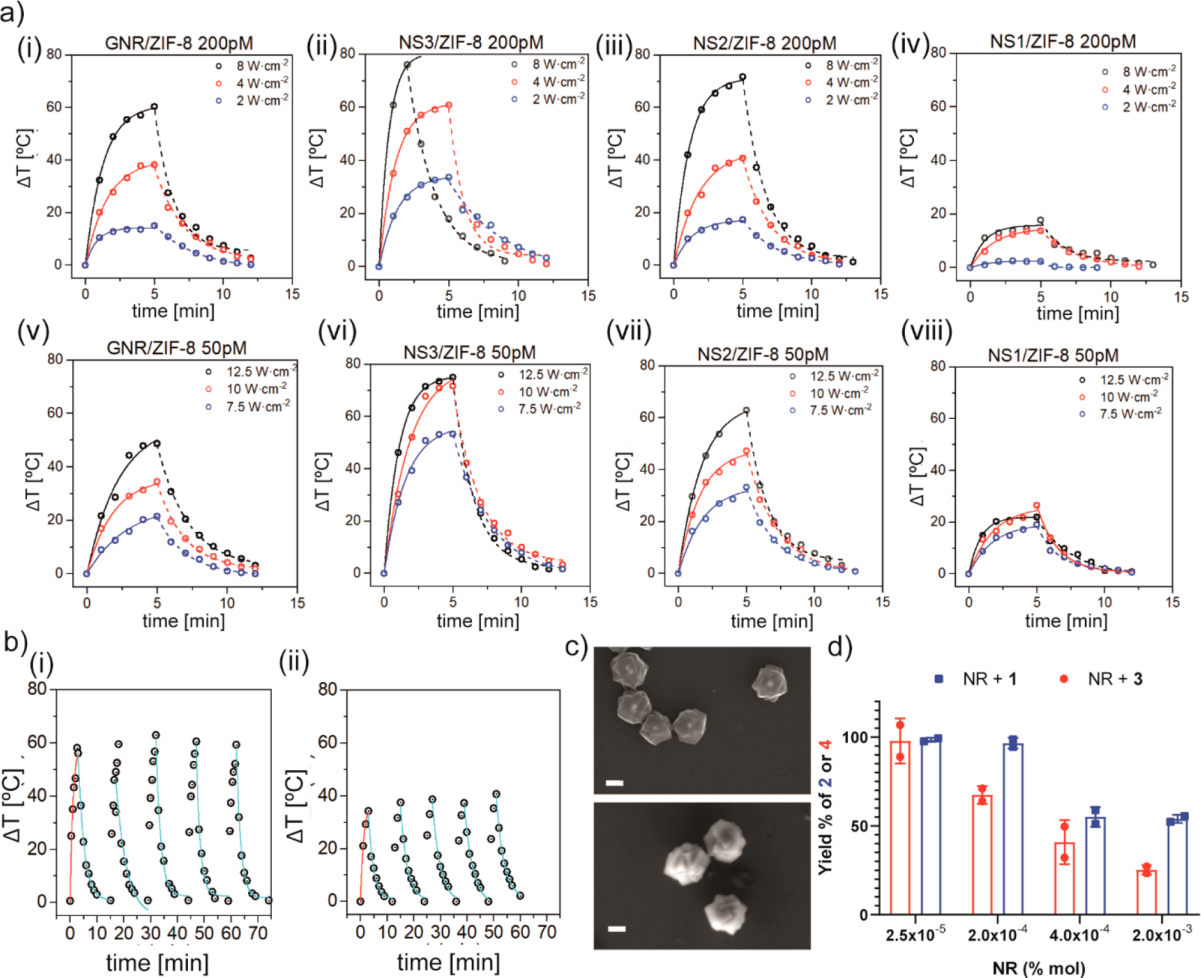
(a) Temperature-irradiation time profiles for NRs dispersed in water at 0.2 nM or 0.05 nM (GNR/ZIF-8 (i) and (v), NS3/ZIF-8 (ii) and (vi), NS2/ZIF-8 (iii) and (vii); NS1/ZIF-8 (iv) and (viii)). Solid lines represent simulation curves for heating (Box–Lucas function), and dashed lines represent simulation curves for cooling (exponential) using the fitting parameters shown in Table S2b). Heating/cooling cycles Δ*T* versus irradiation (NIR, 808 nm) exposure (*t*) for 8 W·cm^–2^ (NRs dispersed in water at 0.2 nM) and 7.5 W·cm^–2^ (NRs dispersed in water at 0.05 nM) applied during 3 min. Red lines represent simulation curves for heating (Box–Lucas function) and blue line represent simulation curves for cooling (exponential). (c) Representative SEM images of the NS2/ZIF-8 particles after irradiation with 808 nm laser at for 8 W·cm^–2^ for 3 min. Scale bars correspond to 200 nm. (d) Yield of **2** (blue bars) and **4** (red bars) after NIR treatment (8 W·cm^–2^ for 2 min) of a water suspension of substrate **1** or **3**. NRs dispersed in water (0.2 nM and 0.04 pmol) with different NR/substrate molar ratio.

To investigate the photothermal behavior of the designed nanocomposites, aqueous solutions of our NRs were irradiated with different doses (NIR irradiation setup is shown in Figure S10), varying irradiance (2, 4, 8, 7.5, 10, and 12.5 W·cm^–2^), exposure time (2, 5, or 10 min), and NR concentration (200 and 50 pM) (Figures 3a,b and S11 and Table S2). Among all the NRs evaluated, NS2/ZIF-8 was chosen for the *in vitro* experiments due to its heating/cooling profiles (consistent heating and cooling rates for the NRs ∼ 0.5 min^–1^ using various conditions, Table S2). Despite the bulk solution achieving a maximum temperature of ∼60–70 °C, the NRs remain colloidally stable, without any sign of aggregation (Figure S12). Electron microscopy confirmed that the integrity of the NR remains intact after NIR treatment (Figures 3c and S12), which contrasts with surfactant-stabilized NSs that do not sustain repeated illumination cycles and gradually lose their heating capability (Figure S13a), likely due to a heat-induced aggregation. This can be prevented by prestabilizing the NSs with fetal bovine serum (FBS; Figure S13b). However, our NRs exhibit a different heating profile compared with that of both surfactant- and FBS-stabilized NSs (Tables S2 and S3). Although using equivalent experimental parameters (0.2 nM particles, NRs or NSs in water, 8 W·cm^–2^), the maximum bulk solution temperature is very similar among the samples, the heating rate for the NS2/ZIF-8 nanoreactor (∼0.5 min^–1^) is significantly damped compared with that for the NSs (∼0.8–1.0 min^–1^, Table S3). This is in agreement with previous work with core–shell gold-silica nanoparticles indicating that the thermal dissipation upon resonant illumination changes with the thermal conductivity of the silica shells.^40^ In the case of our particles (NRs), the intrinsically low thermal conductivity of the ZIF-8 shell compared to that of water^41^ leads to a higher thermal confinement (of the most importance for the foreseen application) and thus a lower heating rate than in the case of surfactant- or FBS-stabilized NSs. For instance, while in the case of the NSs, three irradiation cycles require less than 35 min (Figure S13), an analogous experiment with NRs requires ∼45 min (Figure 3b(i)). Cooling to room temperature (RT ∼ 22 °C) for either NRs or NSs presented similar rates (∼0.5 min^–1^).

With the optimal NRs (NS2/ZIF-8) at hand, we then tested their potential as photothermal catalysts in the thermocyclization reactions indicated in Figure 1b. We chose this type of transformation for several reasons: (i) to demonstrate that it is feasible to carry out abiotic nucleophilic substitutions in biological media and inside living cells; (ii) the requirement of heating for performing the reaction; (iii) the formation of a cyclic fluorogenic product that allows a straightforward quantification (calibration curves in Figures S14 and S15) and, eventually, a fluorescence readout in cells; (iv) and the associated release of leaving groups, which might be useful to produce colored or bioactive products. The conversion of substrates into fluorogenic products was quantified (calibration curves in Figures S14 and S15) using a thermostatic bath at 90 °C, 4 h, as described in the Supporting Information.

First, we tested the loading capacity of our NRs, for which substrate **1** was encapsulated into the core–shell NS2/ZIF-8 particles before the PMA stabilization step (NR@**1**). Using NTA quantification and the corresponding calibration curve, we calculated ∼1.33 × 10^5^ molecules of substrate **1** per particle (i.e., 7.7 × 10^–4^ mol % NR; see details about the quantification of substrate loaded in Supporting Information Section S6). After PMA coating to allow compatibility with water,^23,36^ a suspension of NR@**1** particles (0.2 nM particles) in water was irradiated with the NIR setup (5 min, 8 W·cm^–2^). We observed bulk heating of the mixture (*T*_max_ ∼ 76 °C) and the formation of **2** (46% yield, Figure S16). To quantify the products after any treatment, unless otherwise specified, particles were precipitated by centrifugation (4000 RCF, 30 min), and the probe’s concentration was determined in the supernatant using fluorescence calibration curves (Figures S14 and S15).

We also tested the photothermal performance of the NRs by externally adding substrate **1**, i.e., without previously embedding the reactant within the nanostructures. Therefore, pyridine **1** was mixed in water with the NRs, i.e., NR + **1**, so we obtained the same NR/**1** mole ratio (i.e., 7.7 × 10^–4^ mol % NR) as in the previous case, and the suspension was irradiated for 5 min (8 W·cm^–2^). Gratifyingly, using the fluorescence calibration curve, we calculated a yield of product **2** of 38% (Figure S16a), quite close to that obtained when the substrate was preinserted in the MOF. The slightly lower value with respect to the use of NR@**1** is somewhat expected, since in the case of NR + **1** substrates must access into the plasmonic core NS and undergo the conversion.

Importantly, control experiments using bulk solution heating and the same concentrations of substrate and NRs used above carried out by immersing the samples in a thermostatic bath (5 min, 80 °C) revealed no reaction (Figure S16b, Table S4). This confirms that it is not the temperature of the bath but rather the local heating near the plasmonic NS2 what is triggering the reaction.

Once we optimized the NR photothermal properties and demonstrated that they could work as NIR-gated reactors for promoting the designed nucleophilic substitutions, we tested different NR-to-substrate **1** molar ratios (mol % of NR; in all these test tube experiments, we kept the concentration of NRs constant (∼0.2 nM) and change the concentration of substrates). As expected, using a higher proportion of NR, i.e., decreasing %NR, and equivalent irradiation conditions (2 min, 8 W·cm^–2^), we obtained a 98% yield of **2** (Figure 3d, blue bars). Importantly, control experiments irradiating either ZIF-8 particles lacking the NSs (equivalent to our NRs but without the plasmonic core) and/or the substrate alone, showed virtually no conversion (Table S4).

Notably, by using FBS-stabilized NS2, we observed very poor conversions (Table S4), likely due to the combination of two facts: (i) limited access of substrates to the heated FBS-stabilized NS particles and (ii) poorer thermal confinement in FBS-stabilized NSs than in our NRs. This result confirms the relevance of the microporous shell to allow a suitable accumulation of the reactants near the NIR-heated active site. In all these controls, equivalent experimental conditions (irradiation, particle concentration, and mol % particle) were used, as summarized in Table S4. The reaction can also be extended to other precursors, like coumarin derivative **3**, which in addition to cyclized product **2**, releases fluorescent coumarin **4** (Figure 3d, red bars).

Taking advantage of the photostability of our NRs, we inspected their reusability to produce **2**, using two different approaches (Table S5). First, we confirmed that the NRs can be used, washed by precipitation, and reused in up to five independent reactions leading to virtually equal yields in all the assays (Figure S17a). Second, we observed that performing five consecutive irradiations (2 min)/cooling cycles, the conversion of substrate **1** is almost complete (>96% of **2**; Figure S17b). Likewise, with three consecutive irradiations (5 min)/cooling cycles the yield was over 85% (Figure S17c).

Overall, these results confirm that the improved performance of our NRs compared with the NS particles must be related to its core–shell nanoarchitecture, in which the MOF-based shell shields the heated NS (thermal confinement) and plays a critical role to facilitate the access of substrates to the heated core while allowing an appropriate flow of reactants and products.

Having demonstrated and quantified the photoconversion capability of our NRs in water, we next aimed to demonstrate that the photothermal-responsive reactivity can be performed in the complex milieu of a mammalian living cell. To this end, we first carried out cell viability assays (resazurin assay, Figure S18) using HeLa cells supplemented with different concentrations of NRs, FBS-stabilized NSs, substrates (**1** or **3**), and reaction products **2** and **4**. This allowed us to set the concentration of reagents and NIR irradiation conditions compatible with cell viability (24 h incubation, Figure S18); these are 10 μM (up to 24 h incubation) of probes (**1**, **2**, **3**, or **4**) and 50 pM (overnight, 12–18 h incubation) of particles. With respect to the NIR treatment, we observed no toxicity effects using exposure times of 1 or 3 min, and irradiances of 7.5, 10, or 12.5 W·cm^–2^, in cells loaded with NRs, with or without substrate **1**.

Cellular uptake of fluorescently labeled NRs was confirmed by flow cytometry (Figure S19) and confocal microscopy (Figure S20); after 12 h of incubation of cells with the NRs, we observed saturation of the NR uptake (Figure S19). We quantified the average gold content per cell by inductively coupled plasma mass spectrometry (ICP-MS), which allowed us to estimate the average number of NRs per cell (∼335). We also quantified the amount of internalized gold when using FBS-stabilized NSs instead of the NRs, observing a decrease in the intracellular gold content per cell (∼166 NSs). This is likely consequence of the preformed protein corona, which in general leads to decreased particle uptake by cells.^42^

The intracellular thermolytic reactions were performed by using two approaches. In approach A, cells were mixed with NRs (50 pM) containing the encapsulated probes (NR@**1** or NR@**3**), incubated overnight, washed with PBS to remove noninternalized particles, and mixed with fresh cell medium before NIR treatment (Supporting Information Section S4). In approach B, cells were first incubated with NRs (50 pM, overnight) to obtain NR-loaded cells, washed with PBS, and then mixed with substrates (either **1** or **3**, 10 μM in fresh milieu); after 30 min, cells were washed twice with PBS to remove the noninternalized substrates.

**Figure 4:**
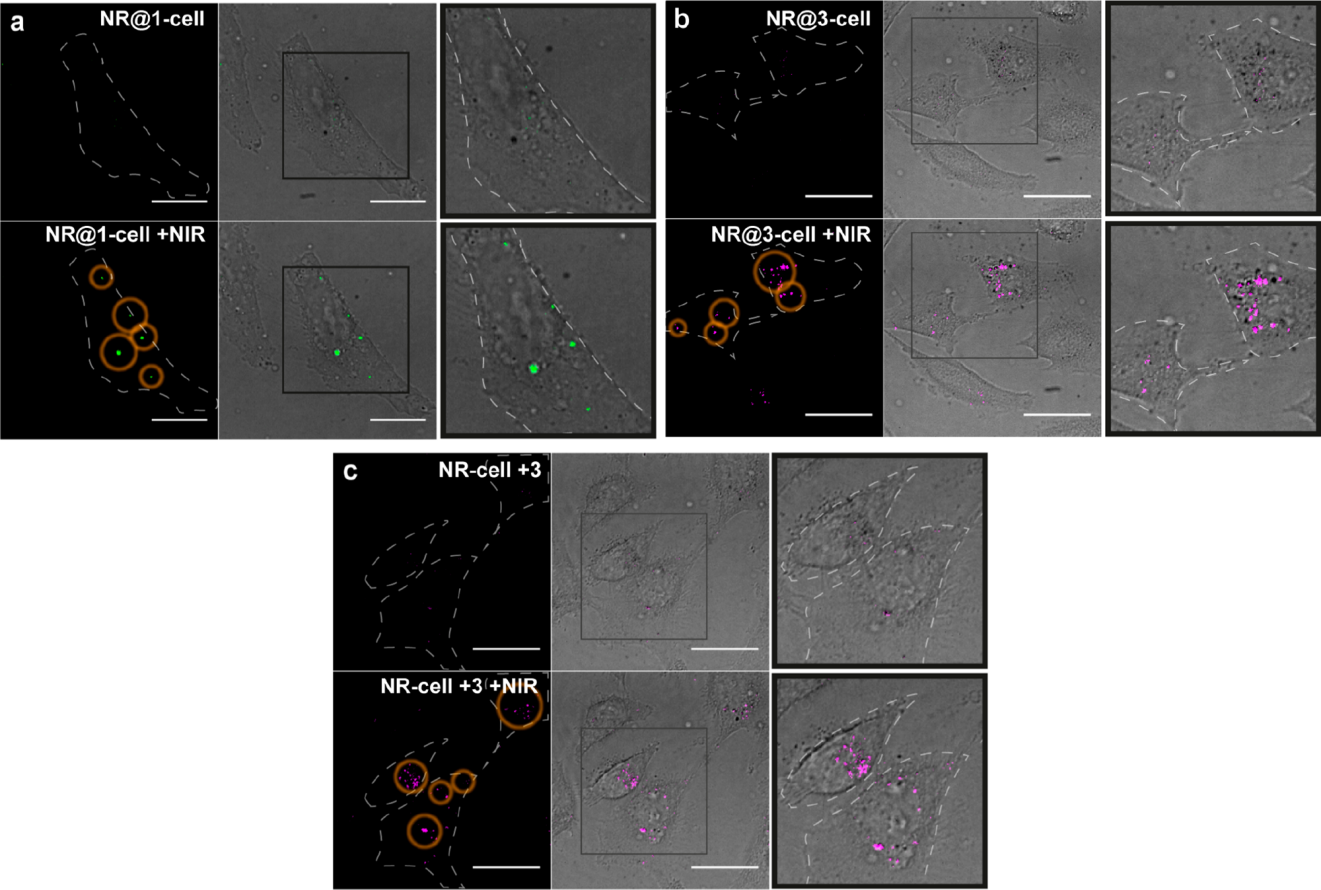
(a–c) Confocal microscopy images before (top) and after (down) NIR-pointer irradiation (∼5–10 s using a 785 nm-NIR pointer with diameter ∼1 μm and ∼5 × 10^5^ W·cm^–2^) of NR-preloaded cells, for which the probes were pre-encapsulated in NRs (a, approach A, NR@**1**; b, NR@**3**) or in which substrate **3** was added to cells preloaded with the NRs (c, approach B). Irradiated regions of interest (ROIs) are marked with orange circles in the fluorescence channels (left images in each panel). The following excitation (ex.)/ emission (em.) wavelengths were used for confocal imaging: 405/450(50) nm (green dots in a) and 405/525(50) nm (pink dots in b and c). Scale bars correspond to 20 μm. Irradiated cells were outlined (discontinued lines); right panels (merged bright field + fluorescence images) are zoomed ROIs (squares in center images).

The irradiations were performed using a 785 nm–NIR pointer (diameter ∼1 μm, circular spot with ∼5 × 10^5^ W·cm^–2^), which allows us to illuminate specific regions of single cells, one after another, that is, with high spatio-temporal resolution. Using the cells preloaded with NR@**1** or NR@**3** (approach A) and short irradiations (∼5–10 s), we observed a buildup of local fluorescence, indicative of the generation of product **2** (green) where specific regions of single cells were irradiated (Figures 4a and S21). The reactivity is even more clear using probe **3**; in this case, we can detect the characteristic fluorescence of coumarin **4** (pink), which is released in the process (Figures 4b and S22).

Using approach B, in which cells are preloaded with “empty” NRs, incubated for 30 min with probe **3** (10 μM), washed, and irradiated with the NIR pointer (785 nm, ∼5 × 10^5^ W·cm^–2^, ∼5–10 s), we also observed a clear intracellular fluorescence increase corresponding to product **4** (Figures 4c and S23). This result confirms the potential of our NRs to function as truly NIR-controlled NRs in which substrates and products can flow. Furthermore, the reaction is not just a simple thermal cyclization but also triggers the release of a secondary functional product which suggests it could be easily adapted for the production of biorelevant products with an impressive spatio-temporal resolution.

**Figure 5:**
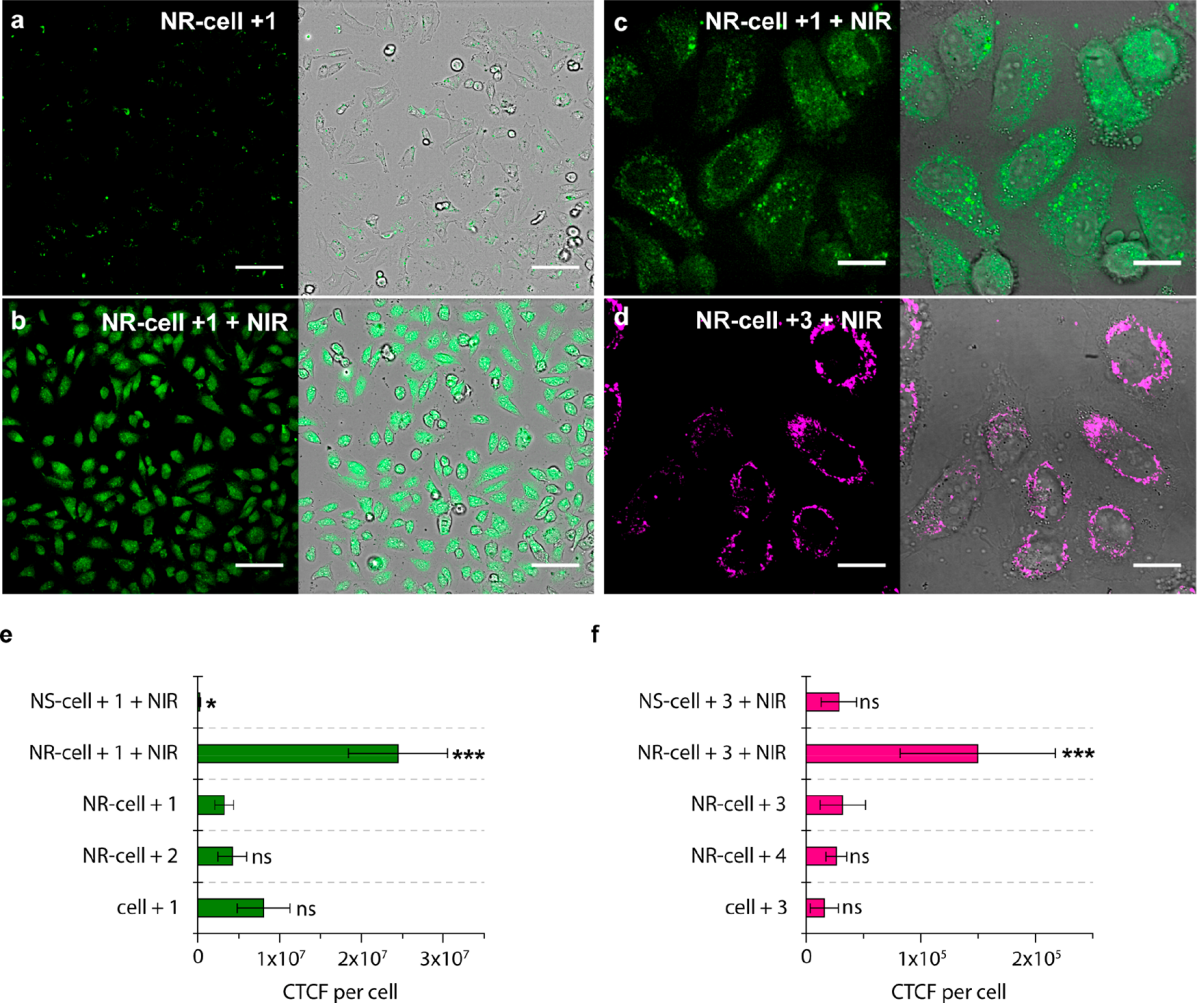
(a) Confocal microscopy image of NR-preloaded cells incubated with probe **1**, without NIR treatment (control). (b–d) Confocal microscopy images of NR-loaded cells with probe **1** (b, c) or **3** (d) after NIR irradiation using the collimated beam system (10 W·cm^–2^, 1 min). In a–d, fluorescence (left a-c, ex./em. = 488/525(50) nm; left d, ex./em. = 540/620(60) nm) and merged bright-field + fluorescence images are shown; scale bars correspond to 100 μm (20×, a and b) or 20 μm (100×, c and d). e, f) Comparison of the intracellular fluorescence (corrected total cell fluorescence – CTCF per cell) generated in the NIR-triggered thermocyclization of substrate **1** (e) or **3** (f) achieved by our NR, or by FBS-stabilized NS2; for comparison, controls without NIR treatment and the different probes are included. In all data statistical significance was assessed by one-way analysis of variance (ANOVA) test comparing each group with the corresponding reference (e: NR-cell + **1**; f: NR-cell + **3**; ns, not significant; *p < 0.05; **p < 0.01; ***p < 0.001). All data expressed as mean ± SD, *n* ≥ 10 (at least 10 individual cells were analyzed for each group; 100x confocal fluorescence images).

To further address the potential of our NIR-controlled strategy, we carried out equivalent intracellular reactions using a collimated NIR beam system (10 W·cm^–2^, 1 min), which allows us to synchronously irradiate tens of thousands of cells. We were glad to observe a clear fluorescence after irradiation of cells containing the NRs and probe **1**, which must be associated with the intracellular generation of product **2** (green, Figure 5a–c). In the case of cells treated with probe **3**, we also observed fluorescence typical of product **4** (pink, Figure 5d). As expected, in the absence of the NIR treatments, using either of the two approaches, there is not intracellular fluorescence (Figures S24 and S26). Notably, analogous experiments using cells loaded with FBS-stabilized NS instead of the NRs (Figures S25 and S27) also failed to promote any fluorescence buildup, which is in agreement with the result of analogous experiments carried out in water (Table S4). It should be noted that without NIR excitation the incubation of the probes (substrates **1** or **3**, or even products **2** or **4**) as previously described failed to generate buildup of intracellular fluorescence, both in the absence or presence of intracellular NRs (Figures S24 and S26). In the case of fluorescent products **2** and **4**, this may be seen contradictory considering the clear buildup of intracellular fluorescence using NIR-promoted transformations (Figure 5b–d). However, it also highlights the potential of our approach to photo-promote the intracellular generation of molecules (**2** and **4**), which under the tested conditions (10 μM in fresh milieu during 30 min, followed by washings with PBS and fresh milieu) hardly accumulate inside living cells.

A comparative analysis using the fluorescence readout (corrected total cell fluorescence , CTCF, per cell) confirmed that our NRs drastically outperform FBS-stabilized NSs (Figure 5e,f), leading no doubts about their superiority. Furthermore, control experiments without NIR irradiation using NR-loaded or non-NR-loaded cells, with either substrates (**1** and **3**) or products (**2** and **4**) revealed statistically nonsignificant (ns) values of CTCF per cell (as reference, NR-loaded cells with substrates **1** or **3** and no NIR, were used).

The reasons beyond the excellent performance of the NRs inside living cells must be related to the core–shell nanoarchitecture in which the MOF-based shell plays a critical role to produce thermal confinement, while providing for an efficient efflux of compounds and the accumulation of reactants near the NIR-responsive heated cores.

## Conclusions

We have demonstrated the feasibility of achieving photothermal-promoted chemical reactions in the interior of living cells. The transformation, consisting of an intramolecular nucleophilic substitution (thermocyclization), is possible thanks to the use of purposely designed MOF-based nanocomposites with a plasmonic core nanostar (NS), which generates heat upon NIR irradiation. The microporous nanoshell is critical for favoring an effective concentration of the reactants near the heating source as well as to warrant the required thermal confinement. Our work also represents an example of a NIR-promoted nucleophilic substitution reaction. Overall, our plasmonic nanocomposites behave as excellent and biocompatible NRs that can work inside living cells to promote non-native transformations. Considering the progress in the use of NIR light for *in vivo* applications, our photothermolysis approach might find important applications in light-controlled therapies.

## Materials and Methods

### Nanoreactors (NRs) Preparation

The herein designed and studied NR consists of a plasmonic core NP (gold nanostar, NS; or gold nanorod, GNR) with a microporous ZIF-8 shell. This core–shell NP/ZIF-8 nanocomposite is overcoated with a PMA-based amphiphilic polymer (i.e., poly[isobutylene-*alt*-maleic anhydride]-*graft*-dodecyl) to render the particles colloidally stable in water and cell medium.^23^ NS/ZIF-8 nanocomposites were prepared by following a previously described protocol.^23^ Briefly, CTAB-coated NSs with different tip-to-tip lengths (also CTAB-coated GNRs) were first prepared (as described in Supporting Information Section S2), and the growth of the ZIF-8 onto these nanoparticles was performed in a second step. To this end, an aqueous solution of zinc nitrate (1 mL, 0.025 M) was added to an aqueous solution of 2-methylimidazole (1 mL, 1.3 M) under magnetic stirring (350 rpm) at room temperature (RT), and immediately after this was added a solution containing the plasmonic particles (1 mL, 2 nM of NS or GNR dispersed in 5 × 10^–4^ M of CTAB). The mixture was stirred for 2 min and left then undisturbed for 3 h at RT. Finally, the particles were isolated, purified, and redispersed in methanol. Next, these as-prepared NS/ZIF-8 or GNR/ZIF-8 particles were functionalized with PMA polymer (either nonmodified or PMA-modified with a TAMRA fluorescent dye) by following a described procedure.^23^ Particles dispersed in methanol were mixed with the solution of the polymer in chloroform in an optimized proportion (600 monomers of polymer per nm^2^ of particle), and the organic solvent was removed in a rotavapor. The dried product was resuspended by addition of NaOH (0.1 M, pH 9) aided by sonication and finally collected and purified with water by centrifugation. The as-prepared NRs dispersed in water were stored at 4 °C in darkness until use. See the Supporting Information for further procedural details.

### NRs Structural Characterization Techniques

Scanning electron microscopy (SEM) images were acquired with a FESEM Zeiss Ultra Plus operated at 3 kV to get surface information or at 20 kV to penetrate deeper into the sample and obtain a greater contrast of the plasmonic nanoparticle into the NR. Transmission electron microscopy (TEM) images were acquired with a JEOL JEM-2010 microscope operated at 200 kV by deposition of the sample on top of a copper grid coated with a layer of carbon. A Biochrom Libra S60 UV–visible (spectrophotometer was used to record UV–vis absorption spectrum of the NRs. Nanoparticle size and concentration were determined by nanoparticle tracking analysis (NTA), using a NanoSight NS300 (Malvern Instrument Ktd) equipped with a 405 nm laser. The hydrodynamic diameter (*d*_h_) and polydispersity indexes (PDI) of the NRs were determined by dynamic light scattering (DLS) using a Malvern Zetasizer Nano ZSP equipped with a 10 mW He–Ne laser operating at a wavelength of 633 nm and fixed scattering angle of 173°. Zeta potential (ζ) was measured with laser Doppler anemometry (LDA) using the same Malvern’s instrument.

### NIR-Irradiation Setup and NRs Thermoplasmonic Properties

For irradiation experiments, the setup consisted of an 808 nm laser (Lasing, no. FC-W-808A) coupled to a zoom fiber collimator (Thorlabs, no. ZC618SMA-B) to control the spot size as well as irradiate cells homogeneously. A power energy meter (Thorlabs, no. PM100D) with a thermal power head (10W, 25 mm, Thorlabs,no. S425C) was used to measure the output power. A viewing card (Thorlabs, no. VRC4) was used to measure the spot size. To evaluate the thermoplasmonic properties of the NR particles, solutions containing the NRs in water (200 μL, 0.2 nM, placed in the wells of a 96-well plate) were irradiated at different power densities (2–12.5 W·cm^–2^) and for different irradiation times (from 0 to 10 min). By measuring the temperature of the solution after each irradiation condition, Δ*T*–irradiation time curves were obtained. The thermoplasmonic properties of pristine NSs (i.e., CTAB-coated NS2) and FBS-stabilized NS2 were also evaluated for comparison. The photostability of the NRs after irradiation was also investigated. Details of procedures, results, and fitting parameters for heating (Box–Lucas function) and cooling (exponential) simulation curves are given in the Supporting Information.

### General Procedure of the Thermal-Promoted Nucleophilic Substitutions by NRs

Probes **1** and **3** were synthesized by adapting previously reported procedures aimed to derivatize carboxylic acids with thermolabile protecting groups;^43^ both compounds include either thermosensitive esters or carbamates, which are subject of deprotection in neutral conditions only by increasing temperature (∼90 °C). The thermoplasmonic properties of the NRs were used to promote the thermal reaction (nucleophilic substitution) depicted in Scheme S1, leading to the transformation of the nonfluorescent substrates (**1** and **3**) into the corresponding fluorescent products (**2** and **4**, respectively). Full synthesis protocols and characterization of the probes are given in the Supporting Information. For the reaction, an aqueous solution of NRs (200 μL, 0.2 nM) was mixed with the solution of the substrate in a well of a 96-well plate, and the mixture was irradiated (different conditions were studied, cf. Supporting Information). After irradiation, the temperature of the solution was measured (*T*_max_), and the NRs were then precipitated by centrifugation. The generated product in the supernatant was quantified by fluorescence using the corresponding calibration curves. Control experiments with FBS-stabilized NS2 and without catalyst, as well as conventional heating in a water bath were also performed. The reusability of the NRs in successive cycles was also investigated. The data of all studied reaction conditions and reusability potential can be found at in Table S4.

### Cell Preparation, Viability, And Internalization Studies

HeLa cells were cultured in complete DMEM (Dulbecco’s modified Eagle’s medium) under humid conditions at 37 °C and 5% of CO_2_ and passaged after reaching confluency. The viability of cells after exposure to the probes and/or particles (NS or NR) was determined by resazurin assay and used as starting point to define appropriate concentration ranges. ICP-MS was used to quantify the average gold content per cell, allowing us to estimate the number of particles per cell. The internalization of NRs over time was qualitatively monitored by cytometry in a Guava easyCyte BG HT flow cytometer (Millipore), using TAMRA-labeled NRs. Cell fluorescence was recorded in the Orange-G channel (ex. 532 nm, em. 620/52 nm), always counting at least 5000 events. Controls were also performed with cells without NR treatment. Mean fluorescence intensity (MFI) was analyzed to compare the amount of NRs internalized by cells at different times after incubation. Full details are given in the Supporting Information.

### Cell Irradiation Experiments and Confocal Imaging

Two irradiation methods were investigated: (i) highly focused NIR spot (high power densities of a 785 nm laser “pointer” with diameter ∼5 μm, as in classical optical tweezers)^44^ for single-cell experiments (exposure times ∼5–10 s) and (ii) illumination of thousands of cells with a large diameter (∼0.65 cm; low power density) collimated NIR beam, for which we used a 808 nm laser, for large-area irradiation aimed to achieve intracellular conversion of substrates into products (optimal irradiation conditions: 10 W·cm^–2^, 1 min). Confocal imaging experiments with living cells were performed in μ-Slide 8 well-ibiTreat chambers (1 cm^2^/well, Ibidi, Germany, no. 80826). Images were captured on an Andor Dragonfly spinning disk confocal system mounted on a Nikon TiE microscope equipped with a Zyla 4.2 PLUS camera (Andor, Oxford Instruments) and an OKO-lab incubator to keep cells at 37 °C during all the experiment. Images were acquired with different magnification objectives (20×, 60×, and 100×) and different ex./em. channels (details in the Supporting Information). All the images were processed with ImageJ.

## Supporting Information Available

Additional data on synthesis and characterization of materials (nanoparticles and probes), full experimental methodology (PDF)
