# Supplementary material for: Plasmonic-Assisted Thermocyclizations in Living Cells Using Metal–Organic Framework Based Nanoreactors
Source: ACS Nano. 2021 Oct 18;15(10):16924–33. doi: 10.1021/acsnano.1c07983 (PMC8552491; doi:10.1021/acsnano.1c07983)
Supplement: Supplementary file 1 — nn1c07983_si_001.pdf [file nn1c07983_si_001.pdf]

Plasmonic-Assisted Thermocyclizations in Living Cells Using Metal-Organic Framework-Based Nanoreactors

Carolina Carrillo-Carrión,[†#] Raquel Martínez,[†#] Ester Polo,[§#] María Tomás-Gamasa,[⊥] Paolo Destito,[⊥] Manuel Ceballos,[†] Beatriz Pelaz,[⁋] Fernando López,[⊥‡] José L. Mascareñas,[⊥]* Pablo del Pino[†]*

[†] Centro Singular de Investigación en Química Biolóxica e Materiais Moleculares (CiQUS), Departamento de Física de Partículas, Universidade de Santiago de Compostela, 15782 Santiago de Compostela, Spain.
[§] Centro Singular de Investigación en Química Biolóxica e Materiais Moleculares (CiQUS), Departamento de Bioquímica y Biología Molecular, Universidade de Santiago de Compostela, 15782 Santiago de Compostela, Spain.
[⊥] Centro Singular de Investigación en Química Biolóxica e Materiais Moleculares (CiQUS), Departamento de Química Orgánica, Universidade de Santiago de Compostela, 15782 Santiago de Compostela, Spain.
[⁋] Centro Singular de Investigación en Química Biolóxica e Materiais Moleculares (CiQUS), Departamento de Química Inorgánica, Universidade de Santiago de Compostela, 15782 Santiago de Compostela, Spain.
[‡] Misión Biológica de Galicia, Consejo Superior de Investigaciones Científicas (CSIC), 36080 Pontevedra, Spain.
[#]These authors contributed equally.
Present address for CCC: Instituto de Investigaciones Químicas, Departamento de Química Orgánica, Universidad de Sevilla, 41012 Seville, Spain

SUPPORTING INFORMATION

TABLE OF CONTENTS **Page**

Section	Page
**Synthesis and characterization of the probes**	s2
**Synthesis of PMA-modified NS/ZIF-8 nanocomposites (nanoreactor, NRs)**	s7
**Morphological/structural characterization of NRs**	s10
**NIR-irradiation set up**	s15
**Thermoplasmonic properties of NRs**	s16
**Performance of the particles as nanocontainer *versus* NR**	s20
**Thermal reaction promoted by NRs**	s22
**Photostability and reusability of the NRs**	s24
**Cell studies**	s26
**References**	s37

Synthesis and characterization of the probes

The compounds **1** and **3** were synthesized by adapting previously reported procedures aimed to derivatize carboxylic acids with thermolabile protecting groups;[1] both compounds include thermosensitive esters, which are subject of deprotection in neutral conditions only by increasing temperature (~ 90 ºC, 1:1 MeCN:phosphate saline buffer pH 7.2, 8 h, 10 mM). Thermocyclization of substrate **1** releases an aromatic carboxylic acid upon nucleophilic intramolecular displacement by the pyridine group (product **2**). On the other hand, thermocyclization of substrate **3**, which holds a carbamate as leaving group, undergoes a concomitant decarboxylation reaction delivering an amine moiety, in this case the fluorogenic coumarin **4**, in addition to the cyclization product **2**. The fluorogenic products **2** and **4** (see Figures S1 and S2), resulting from the thermocyclization reactions depicted in Figure 1, were used herein to assess the efficiency of the thermal reactions (yield of the cyclized product **2** and/or release of **4**) in water and inside living cells. Scheme 1 shows the general synthetic route followed to produce both substrates **1** and **3**.

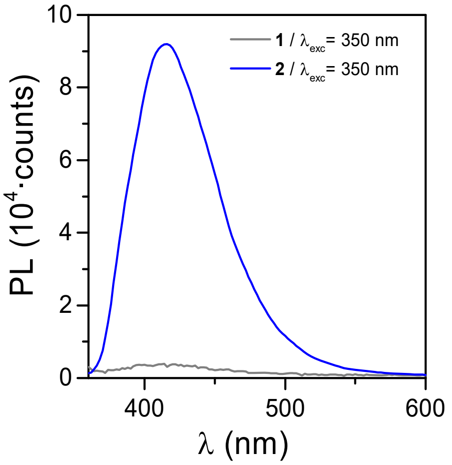

Figure S1. Photoluminescence (PL) spectra of substrate **1** (20 µM, water) and the expected cyclized product (**2**) under excitation at λ_{exc} = 350 nm.

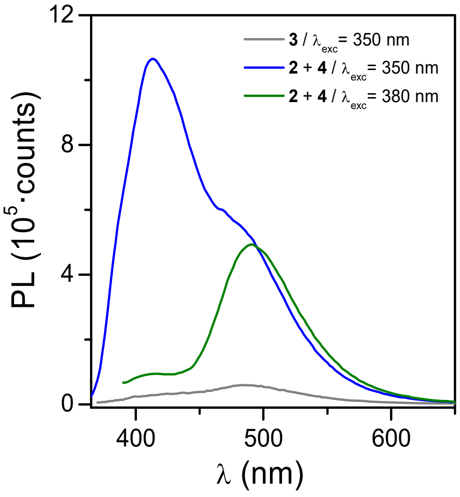

Figure S2. Photoluminescence (PL) spectra of substrate **3** (50 µM, water) under excitation at λ_{exc} = 350 nm, and the cyclized product **2** plus the released product **4** under different excitations.

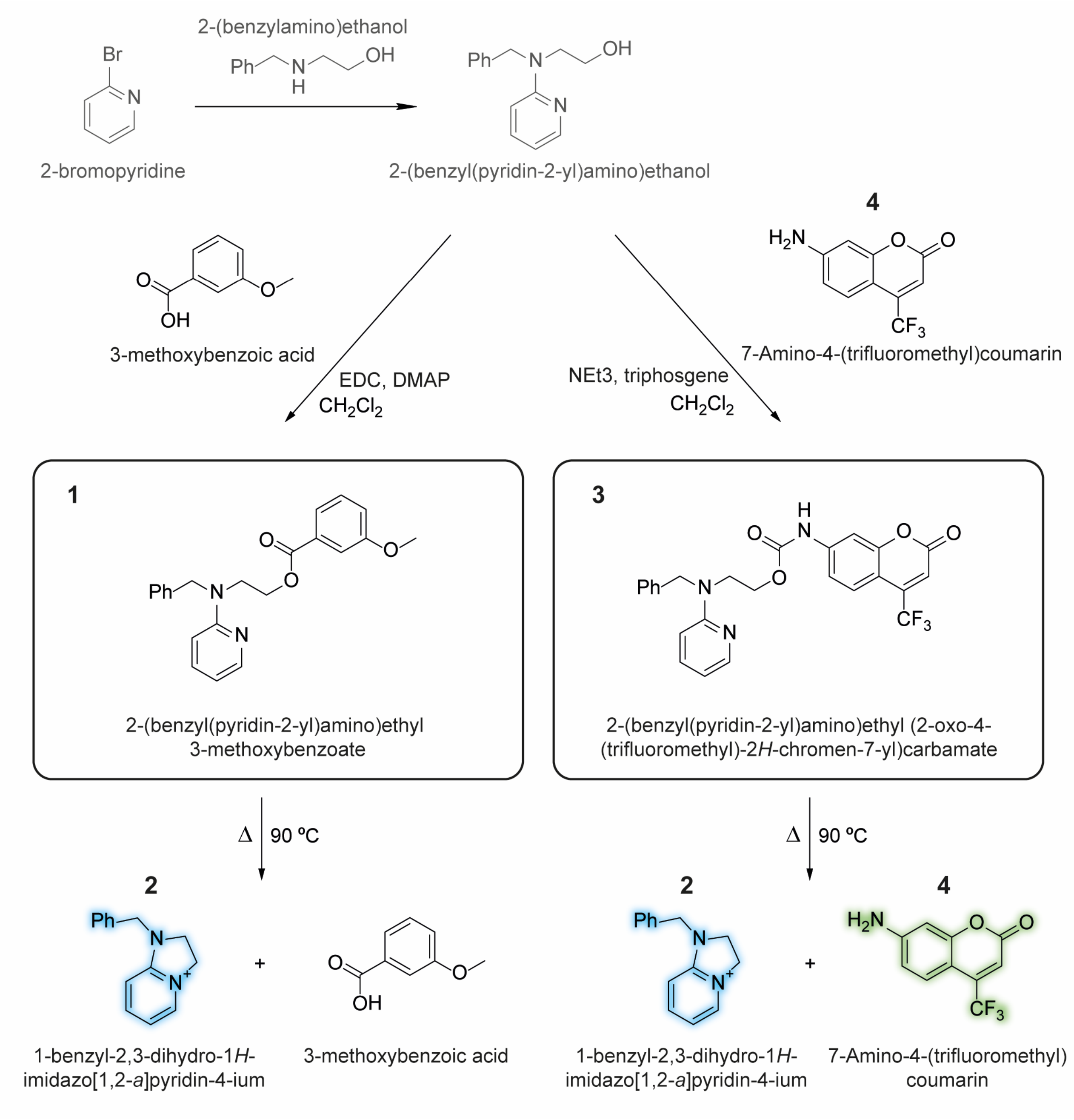

Scheme S1. Diagram of the synthetic method to produce substrates **1** and **3** accompanied by the corresponding thermal-promoted thermocyclization reactions. EDC stands for N-(3-dimethylaminopropyl)-N′-ethylcarbodiimide; CDI stands for 1,1-carbonildiimidazol; DMAP stands for 4-dimethylaminopyridine.

General information for synthesis experiments: Synthetic preparative procedures were performed under an atmosphere of dry nitrogen using vacuum-line and standard Schlenk techniques, unless otherwise indicated. The solvents for organic synthesis were of reagent grade unless otherwise noted. Dry solvents were directly purchased from Aldrich and used without further purification. Water was deionized and purified on a Millipore Milli-Q Integral system.
Chemicals were purchased from Sigma Aldrich, Alfa Aesar, Fluka or Acros Organics and used without further purification.
The abbreviation "RT" refers to reactions carried out approximately at 22 °C. Thin layer chromatography (TLC) was performed on Merck 60 (silica gel F254) plates and components were visualized by observation under UV and / or by using staining solutions of $KMnO_4$ or *p*-anisaldehyde and heating. Chromatographic purification of products was accomplished using flash column chromatography on Merck Geduran Si 60 (40 – 63 µm) silica gel (normal phase). Concentration refers to the removal of volatile solvents *via* distillation using a rotary evaporator Büchi R-210 equipped with a thermostated bath B-491, a vacuum regulator V-850, and a vacuum pump V700, followed by residual solvent removal under high vacuum.
^{1}H NMR, ^{13}C NMR and ^{19}F NMR (300 MHz and 75 MHz, respectively) spectra were recorded at room temperature on a Varian Mercury 300 MHz spectrometer. The spectra were calibrated to the residual solvent peak, if possible. Multiplicities are abbreviated as follows: s = singlet, d = doublet, t = triplet, m = multiplet, and combinations of these. NMR spectra were analyzed using MestreNova© NMR data processing software (www.mestrelab.com). The chemical shifts (δ) are given in ppm and the coupling constants (*J*) in Hz.
UV measurements were performed using a Jasco V-670 spectrometer.
Fluorescence measurements were performed using a Horiba FluoroMax®-3 Spectrophotometer with the following settings: increment 1.0 nm, averaging time 0.2 s, excitation slit width 2.0 nm, emission slit width 5 nm, using 1 cm quartz cells. PMT voltage 620 V.

Synthesis of 2-(benzyl(pyridin-2-yl)amino)ethan-1-ol:

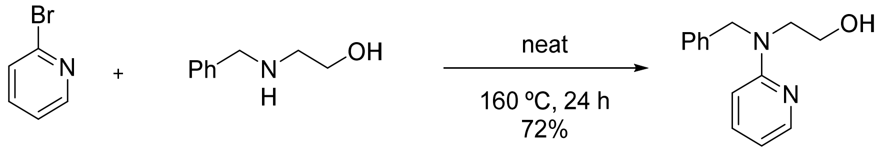

Following a slightly modified version of the reported procedure,[2,3] a mixture of 2-brompyridine (0.602 mL, 6.329 mol, 1 eq.) and 2-(benzylamino)ethan-1-ol (1.800 mL, 12.66 mol, 2 eq.) was heated at 160 °C for 24 h. Upon completion, the reaction mixture was allowed to cool to ambient temperature, diluted with dichloromethane (50 mL) and washed carefully with Na_2CO_3 (sat. solution, 80 mL), followed by brine (50 mL) and H_2O (50 mL). The organic phase was dried over anhydrous Na_2SO_4, filtered and concentrated. The obtained oil was was purified by silica gel column chromatography using 0.5-4% MeOH:CH_2Cl_2 as eluent to give 2-(benzyl(pyridin-2-yl)amino)ethan-1-ol[2] (1.041 g, 4.557 mol, 72%) as a brown oil that underwent crystallization at 25 °C.

^{1}H NMR (300 MHz, $CDCl_3$) δ 8.03 (dd, *J* = 5.1, 1.9 Hz, 1H), 7.40 – 7.06 (m, 6H), 6.67 – 6.32 (m, 2H), 5.73 (broad s, 1H), 4.63 (s, 2H), 3.75 (m, 4H). ^{13}C NMR (75 MHz, $CDCl_3$) δ 158.4, 146.7, 137.6, 137.3, 128.3, 126.1, 126.1, 112.1, 106.4, 61.7, 52.9, 51.9. LRMS (m/z, ESI): 229.12 (M+1).

Synthesis of 2-(benzyl(pyridin-2-yl)amino)ethyl 3-methoxybenzoate (1):

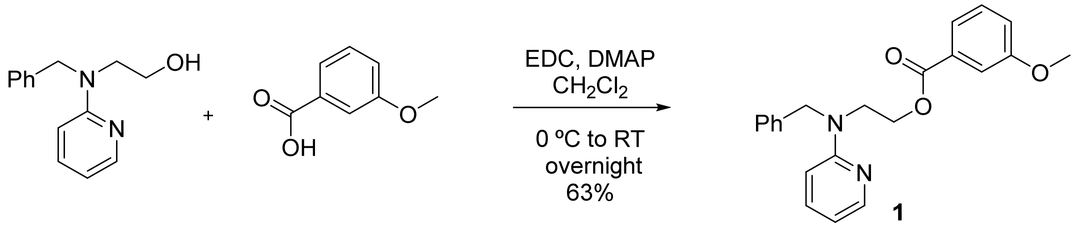

To an ice-water bath cooled solution of 3-methoxybenzoic acid (0.080 g, 0.526 mmol, 1.2 eq.), 1-ethyl-3-(3-dimethylaminopropyl)carbodiimide (EDC) (0.126 g, 0.657 mmol, 1.5 eq.), 4-dimethylaminopyridine (DMAP) (0.013 g, 0.110 mmol, 0.25 eq.) in CH_2Cl_2 (4 mL) was added 2-(benzyl(pyridin-2-yl)amino)ethan-1-ol (0.070 g, 0.438 mmol, 1 eq.) in one portion. The reaction mixture was allowed to reach room temperature and it was stirred overnight. Upon completion, the reaction mixture was diluted with CH_2Cl_2 (15 mL), $NaHCO_3$ (sat. solution, 30 mL) was added, and the organic extracts were washed with brine (30 mL) and H_2O (30 mL). The organic phase was dried over anhydrous Na_2SO_4, filtered, concentrated and purified by silica gel column chromatography using 10% EtOAc:hexane as eluent to give 2-(benzyl(pyridin-2-yl)amino)ethyl 3-methoxybenzoate (**1**, 0.100 g, 0.275 mmol, 63%) as a white solid.

R_f (3:2 hexane:EtOAc) = 0.58

^{1}H NMR (300 MHz, $CDCl_3$) δ 8.18 (ddd, J = 4.9, 2.0, 0.9 Hz, 1H), 7.60 – 7.00 (m, 10H), 6.67 – 6.44 (m, 2H), 4.83 (s, 2H), 4.56 (t, J = 6.0 Hz, 2H), 4.02 (t, J = 6.0 Hz, 2H), 3.81 (s, 3H). ^{13}C NMR (75 MHz, $CDCl_3$) δ 166.4, 159.5, 158.1, 148.0, 138.3, 137.4, 131.4, 129.4, 128.6, 127.0, 126.6, 122.0, 119.6, 113.9, 112.4, 106.2, 63.0, 55.4, 52.7, 47.4. LRMS (m/z, ESI): 363.18 (M+1).

Synthesis of 2-(benzyl(pyridin-2-yl)amino)ethyl (2-oxo-4-(trifluoromethyl)-2H-chromen-7-yl)carbamate (3):

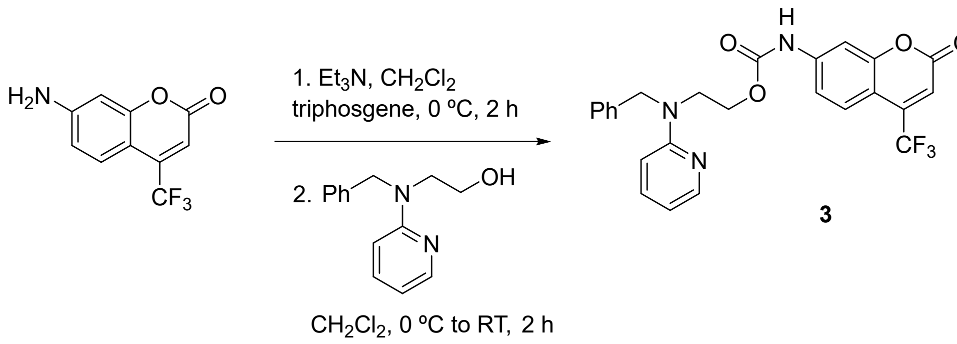

To a solution of 7-amino-4-(trifluoromethyl)coumarin (100.829 mg, 0.44 mmol, 1.0 eq.) and Et_3N (233,14 µL, 0.88 mmol, 2.0 eq.) in anhydrous CH_2Cl_2 (10 mL) at 0 ºC under an Ar atmosphere was added a solution of triphosgene (163.211 mg, 0.55 mmol, 1.3 eq.) in anhydrous CH_2Cl_2 (10 mL). The solution was stirred at 0 ºC for 2 h, and a solution of 2-(benzyl(pyridin-2-yl)amino)ethan-1-ol (100.449 g, 0.44 mmol, 1.0 eq.) in anhydrous CH_2Cl_2 (20 mL) was slowly added at 0 ºC, and the reaction mixture was stirred for 2 h at room temperature. The resulting suspension was concentrated under reduced pressure and the crude was purified by silica gel column chromatography using 20:1 CH_2Cl_2:MeOH as eluent to give 2-(benzyl(pyridin-2-yl)amino)ethyl (2-oxo-4-(trifluoromethyl)-2H-chromen-7-yl)carbamate (**3**) as a yellow oil.

R_f (20:1 CH_2Cl_2:MeOH) = 0.67

^{1}H NMR (300 MHz, CD_3OD) δ 8.07 (ddd, *J* = 5.1, 2.0, 0.9 Hz, 1H), 7.73 – 7.59 (m, 2H), 7.48 – 7.33 (m, 4H), 7.32 – 7.15 (m 4H), 6.77 – 6.52 (m, 2H), 6.38 (s, 1H), 4.84 (s, 2H), 4.41 (t, *J* = 5.8 Hz, 2H), 3.95 (t, *J* = 5.8 Hz, 2H). ^{13}C NMR (75 MHz, CD_3OD) δ 159.8, 153.2, 147.1, 144.8, 144.2, 144.1, 139.3, 138.4, 137.5, 133.0, 128.8, 128.6, 128.2, 127.9, 126.6, 126.4, 126.4, 115.0, 106.9, 101.7, 61.8, 57.3, 51.1. ^{19}F NMR (75 MHz, CD_3OD) δ -65.9 (3F). LRMS (m/z, ESI): 363.18 (M+1).

Procedure for thermal deprotection: Compound **1** or **3** was dissolved in acetonitrile/phosphate saline buffer (1/1, pH 7.2). Deprotection was performed at 90 ºC for 8 h.

Synthesis of PMA-modified NS/ZIF-8 nanoreactors (NRs)

***General information for synthesis experiments**:* All the reagents including zinc nitrate hexahydrate ($Zn(NO_3)_2 \cdot 6H_2O$; Sigma Aldrich #96482), 2-methylimidazole (MeIm; Sigma Aldrich #M50850), and hexadecyltrimethylammonium bromide (CTAB; Sigma Aldrich #H5882) were used as purchased without any purification. CTAB-coated gold nanostars (NSs) and CTAB-coated gold nanorods (GNRs) were prepared by a seed-mediated growth method previously reported,[4] and the PMA-based amphiphilic polymer (*i.e.* poly[isobutylene–alt–maleic anhydride]–graft–dodecyl) was synthesized as described previously.[4] Alternatively, the as synthesized CTAB coating on NSs was exchanged by serum proteins; to this aim, purified CTAB-coated NSs were mixed with fetal bovine serum (FBS) and the mixture was stirred overnight at RT (~ 22 ºC); finally, the FBS-stabilized NSs were collected by centrifugation (10000 RCF, 30 min), washed twice with water, and stored at 4 ºC until use.

The modification of PMA polymer with a fluorophore, in particular here with amino-modified TAMRA (5/6-Tetramethylrhodamine 5/6-carboxamide cadaverine, AnaSpec, #81506) in a ratio of 2 % (*i.e.* dye/PMA monomer ratio of 2/100) was performed according to a procedure already published.[4]

***Synthesis of ZIF-8 nanocomposites**:* These core-shell particles were synthesized according to a previously reported protocol,[4] and consisted in growing a shell of ZIF-8 around CTAB-coated NSs or CTAB-coated GNRs, which act as seeds and in the presence of CTAB as size-controlling and structural-directing agent. First, CTAB-coated NSs with different tip-to-tip length were synthesized and stored at 4ºC in darkness until use. Three different NS, with short tips, medium tips and large tips were prepared. In addition, CTAB coated GNRs were also synthesized as a control and stored at 4ºC in darkness until use.

i) ***Synthesis of NSs with short tips (NS1)***: In a glass flask, 20 mL of an aqueous $HAuCl_4$ solution (0.25 mM) were mixed with 0.03 mL of HCl (1 M) and 1.75 mL of the previously prepared citrate capped-NPs seeds (4 nM),[4] stirring the solution (350 rpm) at room temperature. To this mixture, 0.15 mL of 1 mM $AgNO_3$ and 0.15 mL of 66.67 mM ascorbic acid were added simultaneously. After 10 min, 5 mL of 0.2 M CTAB were added to arrest the NS growth. The solution was kept under stirring for 2 min, and the flask was then immersed in a cold-ice water bath for 10 min. Finally, the NSs were collected by centrifugation (4000 RCF, 10 min), washed twice with water, and finally redispersed in CTAB solution ($5 \cdot 10^{-4}$ M) to ensure the stability of NSs during their storage.

ii) ***Synthesis of NSs with medium tips (NS2)*****:** In a glass flask, 20 mL of an aqueous $HAuCl_4$ solution (0.25 mM) were mixed with 0.03 mL of HCl (1 M) and 1.5 mL of the previously prepared citrate capped-NPs seeds (4 nM),[4] stirring the solution (350 rpm) at room temperature. To this mixture, 0.3 mL of 1 mM $AgNO_3$ and 0.15 mL of 66.67 mM ascorbic acid were added simultaneously. After 10 min, 5 mL of 0.2 M CTAB were added to arrest the NS growth. The solution was kept under stirring for 2 min, and the flask was then immersed in a cold-ice water bath for 10 min. Finally, the NSs were collected by centrifugation (4000 RCF, 10 min), washed twice with water, and finally redispersed in CTAB solution ($5 \cdot 10^{-4}$ M) to ensure the stability of NSs during their storage.

iii) ***Synthesis of NSs with long tips (NS3)*****:** In a glass flask, 100 mL of an aqueous $HAuCl_4$ solution (0.25 mM) were mixed with 0.15 mL of HCl (1 M) and 1.25 mL of the previously prepared citrate capped-NPs seeds (4nM),[4] stirring the solution (350 rpm) at room temperature. To this mixture, 1.5 mL of 1 mM $AgNO_3$ and 0.75 mL of 66.67 mM ascorbic acid were added simultaneously. After 10 min, 25 mL of 0.2 M CTAB were added to arrest the NS growth. The solution was kept under stirring for 2 min, and the flask was then immersed in a cold-ice water bath for 10 min. Finally, the NSs were collected by centrifugation (4000 RCF, 10 min), washed twice with water, and finally redispersed in CTAB solution ($5 \cdot 10^{-4}$ M) to ensure the stability of NSs during their storage.

iv) ***Synthesis of GNRs***: Small gold seeds of nominal diameter around 3 - 4 nm were synthesized. A solution of 0.25mL of $HAuCl_4$, 0.01 M was added to 7.5 mL of CTAB 0.2 M solution at 26 ºC. Then 0.6 mL of freshly prepared 0.01M $NaBH_4$ was added rapidly to the stirring solution (350 rpm) and let 2 min. The solution color changed immediately from yellow to brown. The seed solution remained under stirring for 1 h. The temperature was kept at 26 ºC over all the reaction steps. For GNR formation: 4.25 mL of 0.01M $HAuCl_4$ solution was added to 100 mL of 0.2 M solution of CTAB stirring at 26 ºC. Then 1.23 mL of 0.01 M $AgNO_3$ solution was added, and after 2 min stirring, freshly prepared ascorbic acid solution (0.68 mL 0.1M) was added. After 10 min of stirring, 1.1 mL of the seed solution synthesized as described above, was added and stirring for other 2 min. The final solution was left overnight at 26 ºC in a water bath. The synthesized GNRs were purified twice by centrifugation at 10000 g for 30 min, and finally the pellets were dispersed in $5 \cdot 10^{-4}$ M CTAB solution.

Then, for the formation of the ZIF-8 onto the NSs and GNRs, an aqueous solution of zinc nitrate (1 mL, 0.025 M) was added to an aqueous solution of 2-methylimidazole (1 mL, 1.3 M) under magnetic stirring (350 rpm) at room temperature (RT), and immediately after a solution containing the NSs (1 mL, 2 nM of NSs dispersed in $5 \cdot 10^{-4}$ M of CTAB) was added. The mixture was stirred for 2 min and left then undisturbed for 3 h at RT, observing during this time the gradually appearance of bluish turbidity due to the formation of the NS/ZIF-8 particles. Finally, the particles were collected by centrifugation (4000 RCF, 10 min), washed twice with methanol (MeOH) and redispersed in 1 mL of MeOH. The concentration of particles in this solution was assumed to be 2 nM, taking into account that one NS/ZIF-8 particle was formed per NS as confirmed by SEM. Note that NSs acted as seeds and that under the here described optimized conditions virtually all the ZIF-8 particles contain a single NS in their geometric center (*cf.,* Figures S3, S4, S5). For all the *in vitro* experiments core-shell particles prepared by using CTAB-coated NSs with a tip-to-tip length of *ca.* 80 nm as a core were used.

Post-functionalization of ZIF-8 nanocomposites with PMA polymer: The as-prepared NS/ZIF-8 particles and GNR/ZIF-8 particle were functionalized with a PMA-based amphiphilic polymer (*i.e.,* poly[isobutylene–alt–maleic anhydride]–graft–dodecyl) by following a recently described protocol.[1] Briefly, the NS/ZIF-8 particles dispersed in methanol were mixed with the solution of the polymer in chloroform in an optimized proportion; in particular 600 monomers of polymer per nm^2 of NS/ZIF-8 particle, assuming a spherical particle of diameter ~ 250 - 350 nm, were added, and the mixture was placed in a rotary evaporator. After complete evaporation of the solvent (3:1 MeOH:$CHCl_3$) the dried product was resuspended by addition of NaOH (0.1 M, pH 9) and aided by sonication (1-2 min). The resulting NS/ZIF-8/PMA nanocomposite (or nanoreactor, NR from now) was collected and purified by centrifugation (4000 RCF, 10 min), washed twice with NaOH 0.01M, and finally redispersed in water (2 nM particle concentration). The same post-functionalization approach was carried out with a TAMRA-modified PMA polymer in order to obtain fluorescent-labeled NRs for their following tracing inside cells.

A scheme of the steps involved in the synthesis of these NRs is presented in Scheme S2.

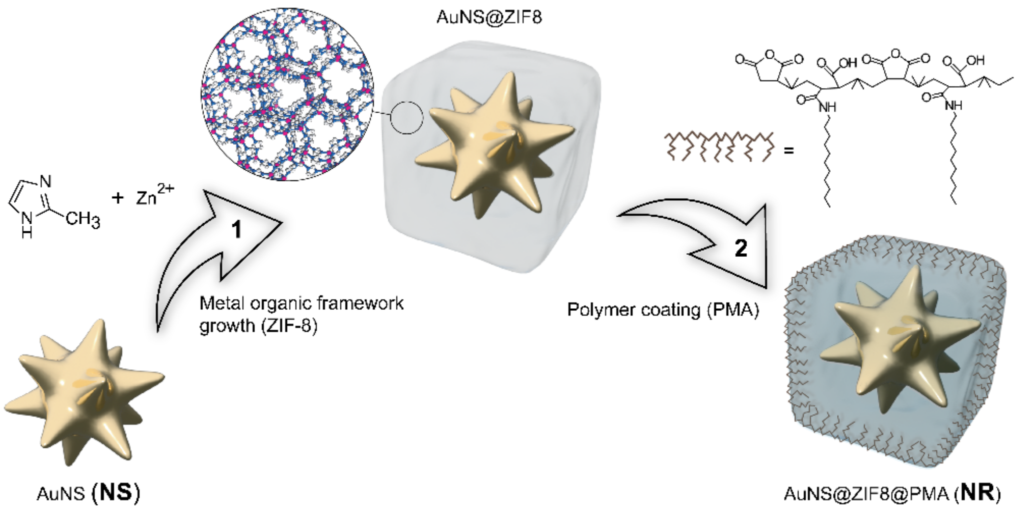

Scheme S2. Schematic illustration of the synthetic procedure of PMA-modified NS/ZIF-8 nanocomposites (NRs).

Morphological/structural characterization of NRs

***Scanning Electron Microscopy (SEM) and Transmission Electron Microscopy (TEM)**:* The size and morphology of NRs was investigated with SEM and TEM (Figure S3, S4 and S5).

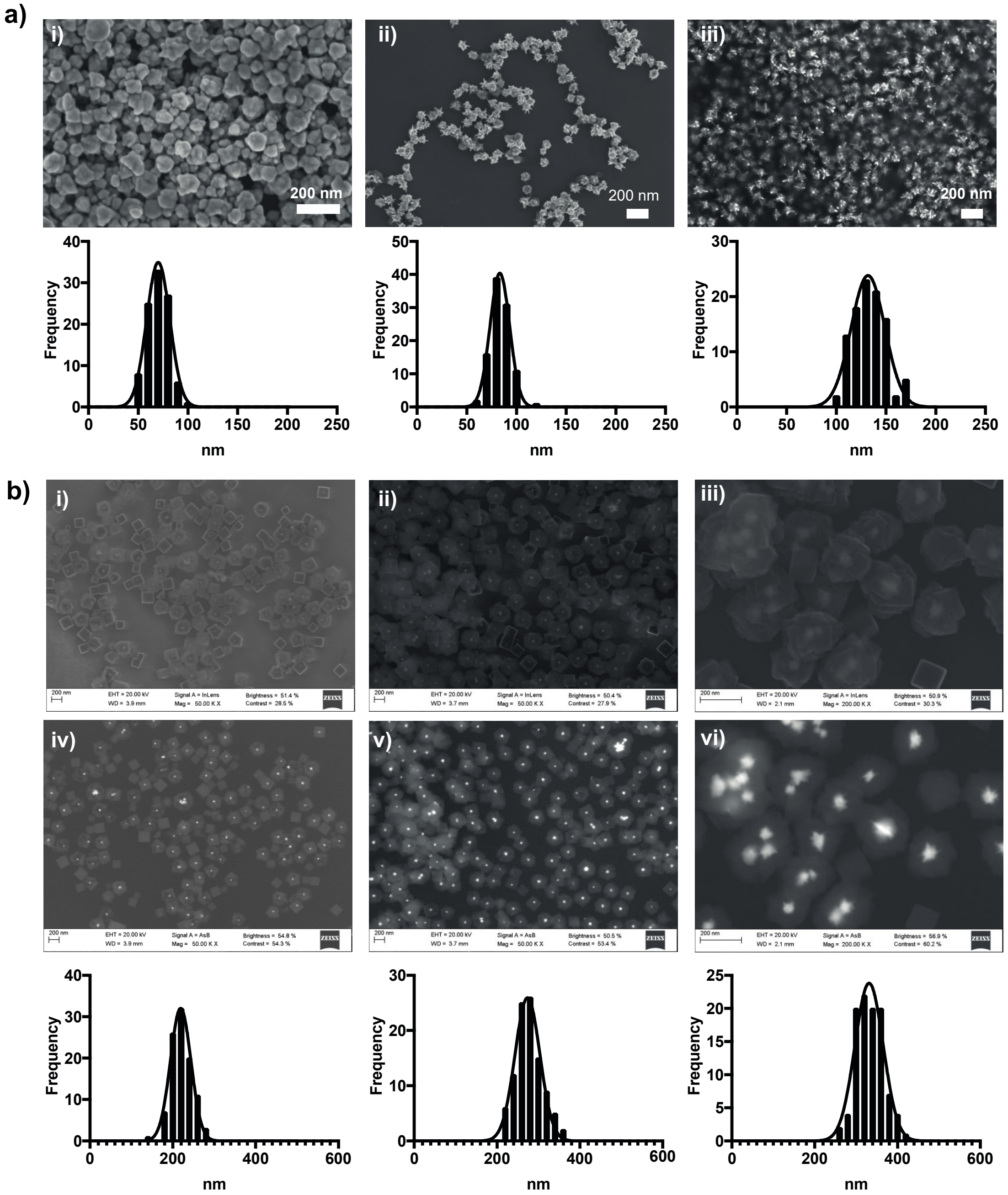

Figure S3. a) Representative SEM images of NS1 (i), NS2 (ii) and NS3 (iii); histogram of the number distribution N of the diameter (*i.e.*, tip-to-tip distance) d_{NS} of the NSs (idealized as spherical particles) as determined from SEM images, d_{NS1} = (70 ± 10) nm, d_{NS2} = (84 ± 10) nm and d_{NS3} = (133 ± 16 nm). b) Representative SEM images of NS/ZIF-8 NRs made from NS1 (i, iv), NS2 (ii,v) and NS3 (iii,vi). Histogram of the number distribution N of the diameter (*i.e.*, vertex-to-vertex distance) d_{NR} of the NRs (idealized as spherical particles) as determined from SEM images, d_{NR1} = (219 ± 25) nm, d_{NR2} = (274 ± 30) nm and d_{NR3} = (331 ± 34) nm. Scale bar corresponds to 200 nm.

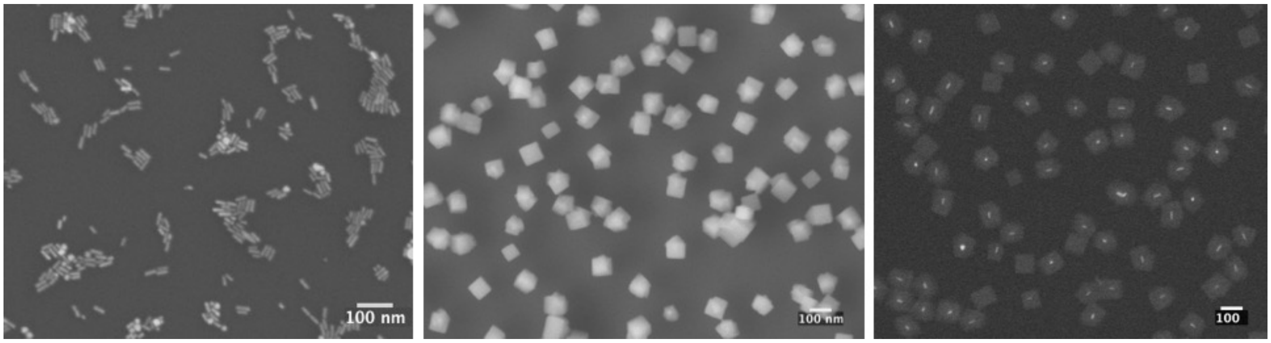

Figure S4. Representative SEM images of GNRs (length (d_l = 40 nm) and width (d_w = 9 nm) and GNR/ZIF-8 nanocomposites.

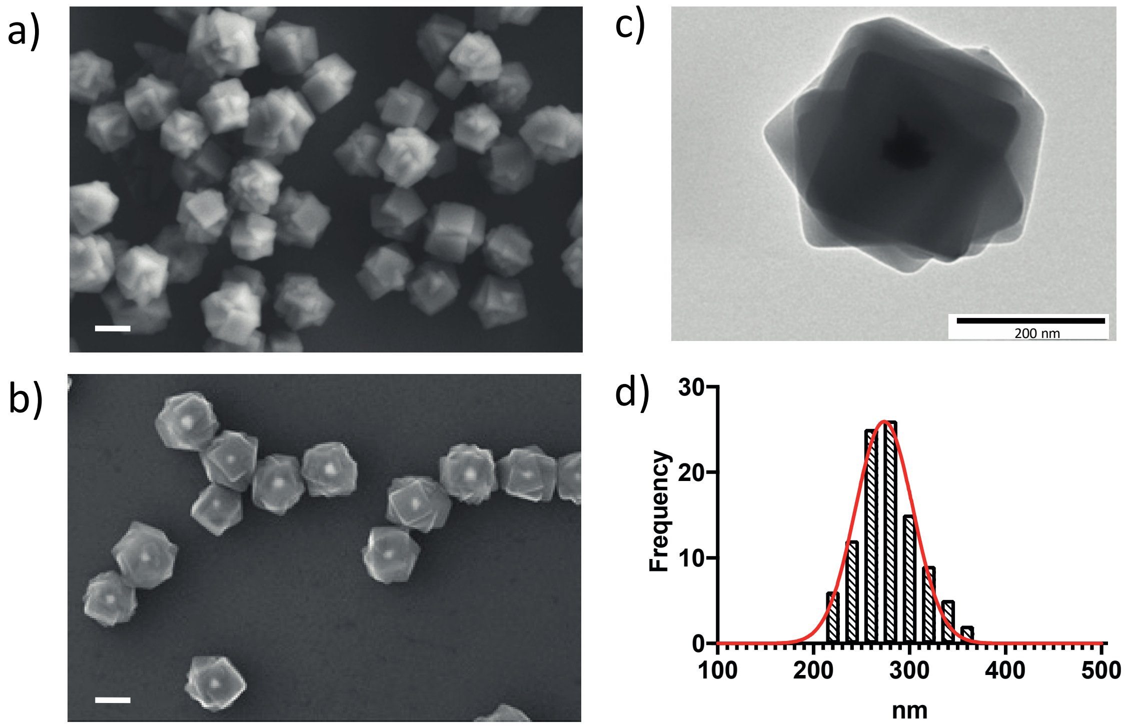

Figure S5. Representative SEM images of the NRs used for the *in vitro* studies taken at different voltages: a) 3 kV; b) 20 kV. c) Representative TEM image of the NR. Scale bars correspond to 200 nm. c) Histogram of the number distribution N of the diameter (*i.e.*, vertex-to-vertex distance) d_{NR} of the NRs (idealized as spherical particles) as determined from SEM images, d_{NR} = (274 ± 30) nm.

***UV/Vis spectroscopy**:* UV/Vis absorption spectrum of the NSs, GNRs and core-shell ZIF-8 nanocomposites in water, which is presented in Figure S6. UV/Vis absorption spectrum of NRs used for *in vitro* studies showed a SPR band centered at 770 nm.

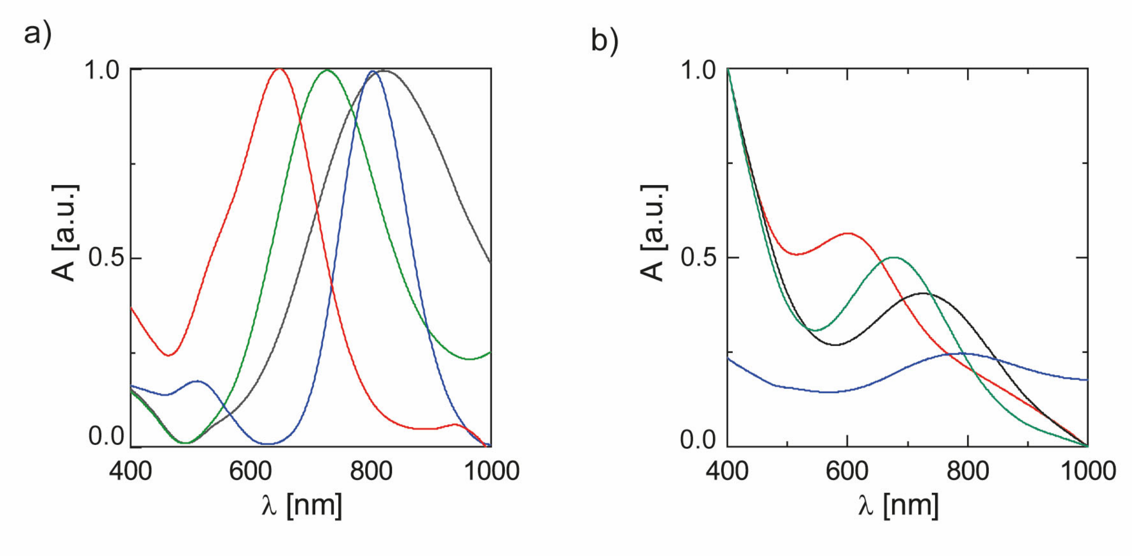

Figure S6. UV/Vis absorption spectra of GNRs (blue line, SPR band centered at 805 nm.), NS1 (red line, SPR band centered at 695 nm), NS2 (green line, SPR band centered at 770 nm) and NS3 (black line, SPR band centered at 820 nm) are shown in the left panel and after ZIF-8 nanocomposite formation in the right panel.

Dynamic light scattering (DLS) and Zeta-Potential (ζ): DLS histograms of the NRs in water and the corresponding hydrodynamic diameter (d_h) values are presented in Figure S7 and Table S1, respectively. DLS measurements of the NRs dispersed in either methanol or water (after PMA functionalization) were performed at different time points (from 0 to 30 days) in order to evaluate the colloidal stability of the NRs in both media (Figure S8). DLS measurements of the NRs dispersed in either water or cell medium (*i.e.* DMEM supplemented with 10 % FBS) were performed at different time points (from 0 to 7 days) in order to evaluate the colloidal stability of the NRs in both media (Figure S9). The ζ -potential of the NRs in water indicated that the NR particles were negatively charged ($\zeta = -27.5 \pm 0.3$ mV) as it was expected due to the carboxyl groups of the PMA polymer.

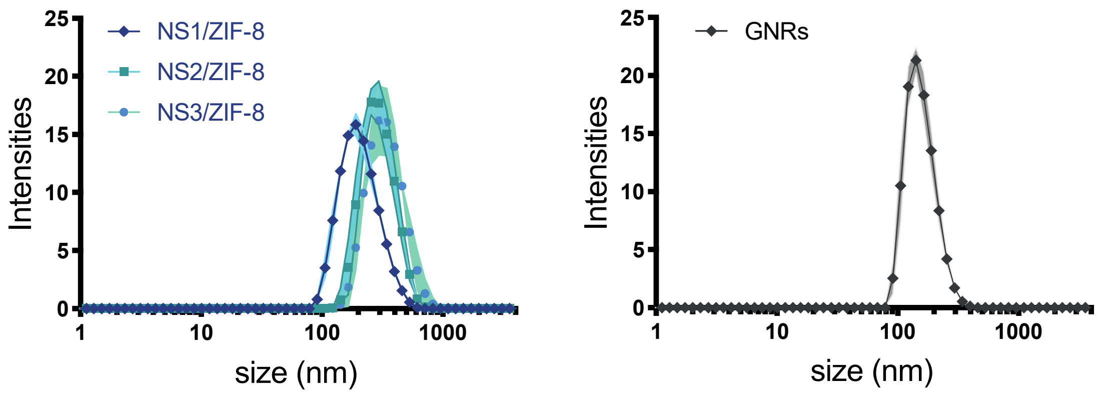

Figure S7. DLS histograms of the NRs coated with PMA (NSs left panel and GNRs right panel) in water**.** Number distributions from DLS data obtained from several repetitions (n=3) of the measurement.

Table S1. Hydrodynamic diameter d_h (mean value ± SD) given as number distributions from DLS measurements of the NRs coated with PMA and dispersed in water. SD values correspond to the standard deviation of the diameter mean value as obtained from several repetitions (n=3) of the measurement. The polydispersity index (PDI) for each sample is also given. Data correspond to the raw data depicted in Figure S7.

Sample	d_h (nm) ± SD	PDI
NS1	214 ± 1	0.19
NS2	301 ± 12	0.09
NS3	347 ± 59	0.14
GNR	159 ± 5	0.10

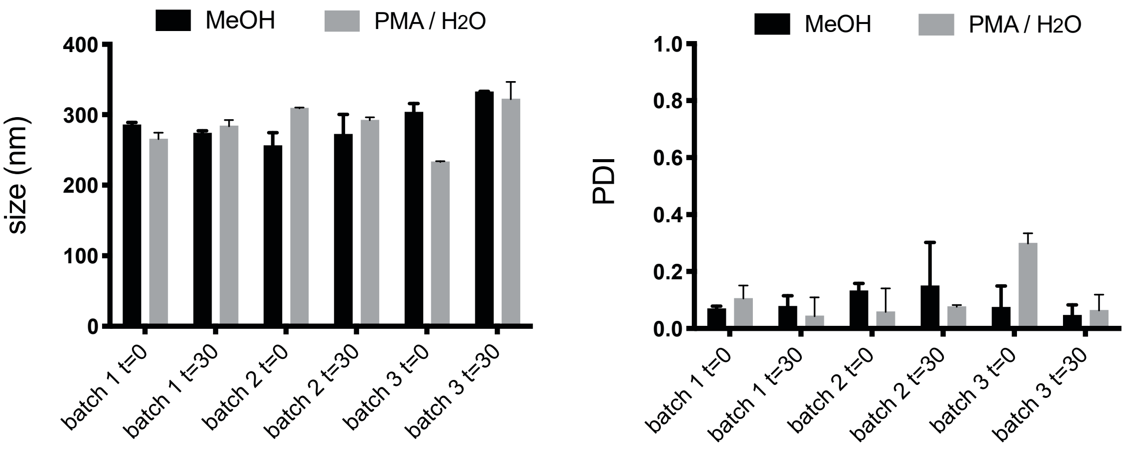

Figure S8. Hydrodynamic diameter d_h (mean value ± SD) given as number distributions from DLS measurements of the 3 different batches of NRs (NS2/ZIF-8) dispersed in methanol and in water after PMA coating at time 0 days and 30 days. SD values correspond to the standard deviation of the diameter mean value as obtained from several repetitions (n=3) of the measurement.

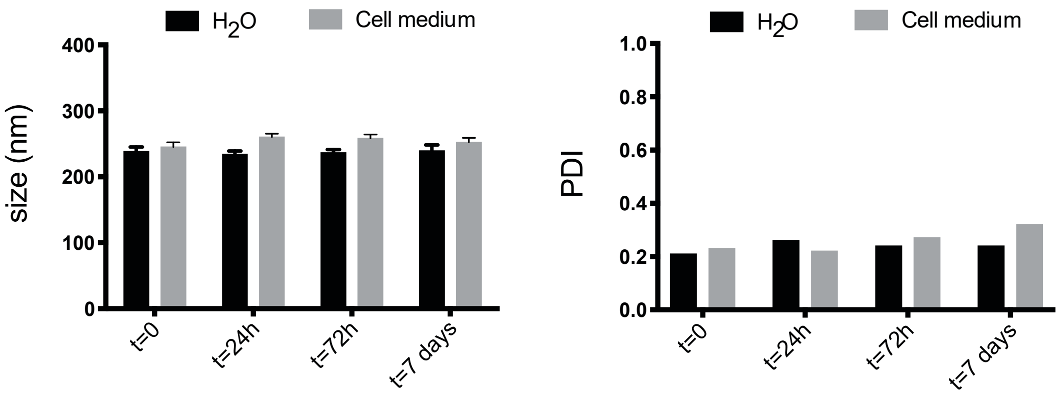

Figure S9. Time evolution of the hydrodynamic diameter d_h (mean value ± SD) given as number distributions from DLS measurements of the NRs dispersed in water or in cell culture medium. SD values correspond to the standard deviation of the diameter mean value as obtained from several repetitions (n=3) of the measurement. The polydispersity index (PDI) for each sample is also given in the right panel.

NIR-irradiation set-up

For laser irradiation experiments, the samples (without or with cells depending on the experiment type as described in each section) were placed in wells of a 96-well plate and were irradiated by using an 808 nm laser (Lasing, #FC-W-808A) coupled to a zoom fiber collimator (Thorlabs, #ZC618SMA-B) to control the spot size as well as irradiate cells homogeneously, *cf.* Figure S10. In order to calculate the intensity in W/cm^2 that cells receive, a power energy meter (Thorlabs, #PM100D) with a thermal power head (10W, 25 mm, Thorlabs, #S425C) was used to measure the output power. To obtain the spot size a viewing card (Thorlabs, #VRC4) was used to see the spot and ImageJ to measure it. Due to the beam is collimated, a homogeneous spot was assumed, and thus the intensity could be calculated just dividing the power by the surface (in cm^2) of the spot. Different time and power densities conditions were investigated as indicated in each experiment.

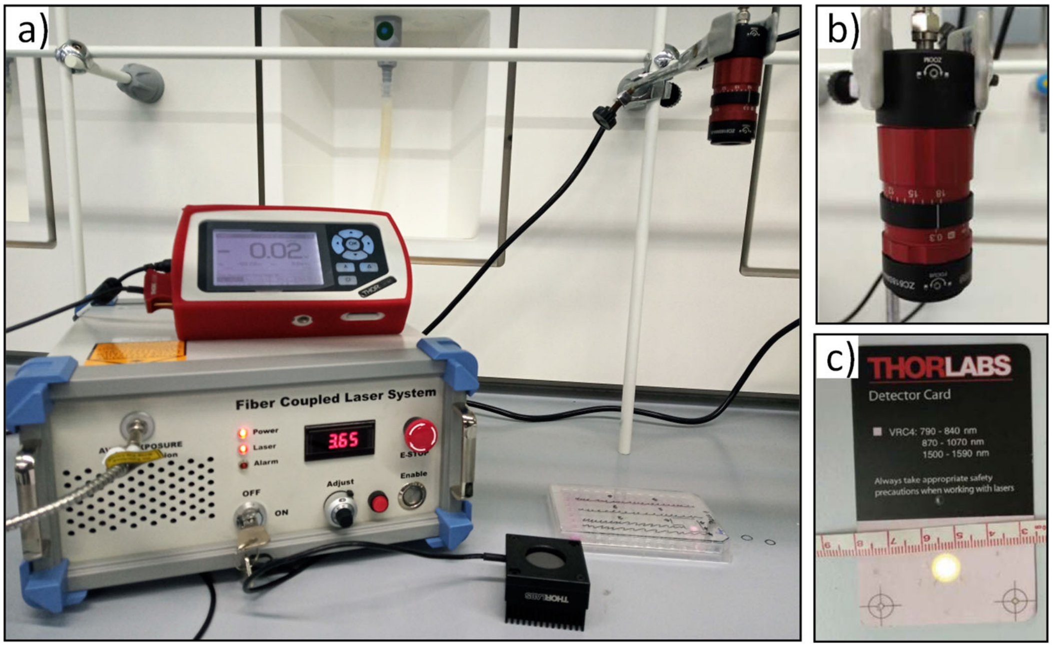

Figure S10. a) Image of the NIR irradiation set-up; b) collimation/zoom system; and c) spot diameter of the beam.

Thermoplasmonic properties of NRs

To evaluate the thermoplasmonic properties of the NR particles, solutions containing the NRs in water (200 µL, at different concentrations, placed in the wells of a 96-well plate) were irradiated with a 808 nm laser at different power densities (2, 4, 8, 7.5, 10 and 12.5 W·cm^{-2}) and for different irradiation times (from 0 to 10 min). The temperature of the solution after each irradiation condition was measured obtaining the ΔT presented in Figure 3a and Figure S11. Water without NRs was used as control showing no obvious change of temperature (*i.e.* only an increase of 2 °C was observed after 10 min of irradiation at 8 W·cm^{-2}). These results indicated that the NRs can rapidly absorb NIR light and efficiently convert the light energy into thermal energy. Moreover, the stability/reproducibility of the photothermal conversion was investigated by performing repetitive irradiation cycles, as shown in Figure 3b. After each irradiation of 3 min at 8 W·cm^{-2} or at 7.5 W·cm^{-2} the cooling profile was recorded and once the solution reached the initial temperature (RT, ~ 22 °C), a new irradiation cycle was applied. No significant change was observed for the maximum temperature in the three laser on/off cycles in the case of the NRs. These results of successive "heating and cooling" cycles demonstrated the good reproducibility of the thermoplasmonic behaviour as well as the photostability of the NRs after irradiation. For comparison the same was done to pristine NSs (*i.e.*, CTAB-coated NSs; Figure S12a), which after just one cycle evidenced aggregation and a weaker thermoplasmonic response than that of NRs. In contrast, FBS-stabilized NSs retained their photothermal capabilities during three consecutive cycles (Figure S12b). The robust photostability of the NR particles was also confirmed by SEM images of the NR after irradiation (*cf.,* Figure S13).

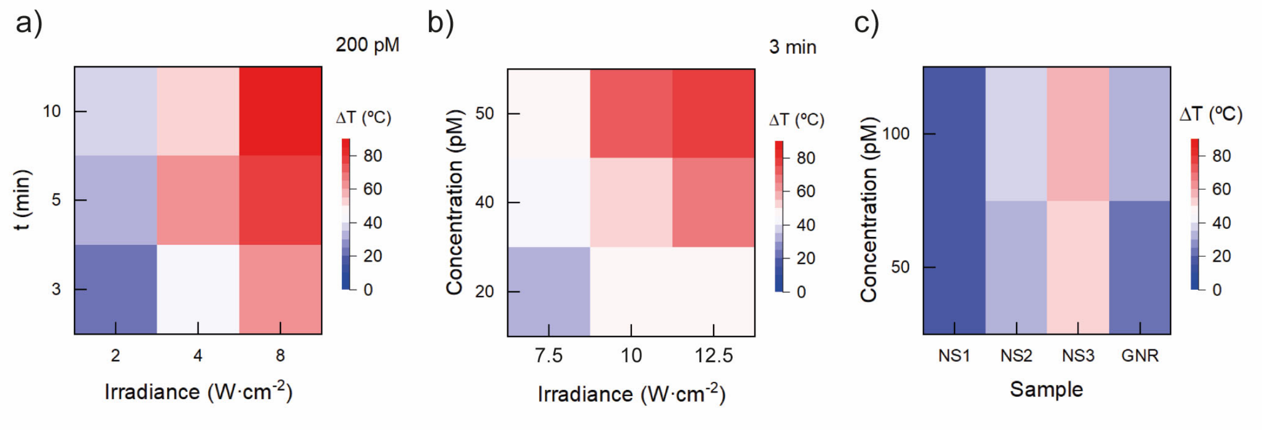

Figure S11. Heatmaps of maximum ΔT after NRs laser irradiation: a) at different laser power densities and different times of 200pM of NS2/ZIF-8 NR; b) at different laser power densities and different NS2/ZIF-8 NR concentrations after 3 min irradiation time; c) at different concentrations and different NRs after 3min irradiation time at 7.5 W·cm^{-2}.

Table S2. Fitting parameters for heating (Box-Lucas function) and cooling (exponential) simulation curves of NRs in water (Figure 3).

**Sample**	**irradiation time t (min)**	**irradiance ($W\cdot cm^{-2}$)**	**fitting curves**					
			**heating / $\Delta T = T_{max}\cdot(1-e^{-b\cdot t})$**			**cooling / $\Delta T = T_0 + A\cdot e^{-b\cdot t}$**		
			**T_{max} (ºC)**	**b (min^{-1})**	***R^2***	**T_0 (ºC)**	**b (min^{-1})**	***R^2***
NS1/ZIF-8 200 pM	5	2	2.52 ± 0.25	0.75 ± 0.24	0.95	0.02 ± 0.02	2.01 ± 0.15	0.99
		4	14.78 ± 0.6	0.6± 0.07	0.99	0.08 ± 0.03	0.4 ± 0.03	0.99
		8	15.81 ± 1.07	1 ± 0.28	0.96	2.15 ± 0.8	0.61 ± 0.14	0.95
NS1/ZIF-8 50 pM	5	7.5	19.35 ± 1	0.58 ± 0.08	0.99	1.06 ± 1.5	0.38 ± 0.07	0.98
		10	26.04 ± 0.26	0.56 ± 0.14	0.97	0.52 ± 0.1	0.65 ± 0.01	0.99
		12.5	21.9 ± 0.26	1.16 ± 0.06	0.99	1.13 ± 0.55	0.65 ± 0.09	0.99
**NS2/ZIF-8 200 pM**	**5**	**2**	**17.21 ± 0.58**	**0.76 ± 0.09**	**0.99**	**0.1 ± 0.5**	**0.41 ± 0.03**	**0.99**
		**4**	**43.75 ± 2.2**	**0.55 ± 0.07**	**0.99**	**0.65 ± 0.65**	**0.5 ± 0.03**	**0.99**
		**8**	**71.19 ± 0.88**	**0.60 ± 0.04**	**0.99**	**2.48 ± 1.1**	**0.61 ± 0.04**	**0.99**
**NS2/ZIF-8 50 pM**	**5**	**7.5**	**33.81 ± 2.26**	**0.55 ± 0.09**	**0.98**	**0.47 ± 0.55**	**0.47 ± 0.03**	**0.99**
		**10**	**47.86 ± 1.51**	**0.63 ± 0.05**	**0.99**	**0.1 ± 0.87**	**0.45 ± 0.03**	**0.99**
		**12.5**	**66.1 ± 0.88**	**0.58 ± 0.02**	**0.99**	**4.67 ± 1.2**	**0.64 ± 0.05**	**0.99**
NS3/ZIF-8 200 pM	5	2	34.11 ± 0.47	0.77 ± 0.03	0.99	2.1 ± 4.6	0.26 ± 0.08	0.98
		4	61.63 ± 0.29	0.85± 0.01	0.99	4.01 ± 1.7	0.93 ± 0.15	0.98
		8	80.37 ± 0.31	1.42 ± 0.02	0.99	1.16 ± 0.93	0.52 ± 0.02	0.99
NS3/ZIF-8 50 pM	5	7.5	56.88 ± 1.85	0.64 ± 0.06	0.99	0.59 ± 0.47	0.38 ± 0.01	0.99
		10	79.77 ± 3.98	0.54± 0.07	0.99	3.81 ± 0.03	0.5 ± 0.04	0.99
		12.5	75.53 ± 0.34	0.94 ± 0.02	0.99	2.1 ± 1.2	0.66 ± 0.05	0.99
GNR/ZIF8 200 pM	5	2	14.19 ± 0.38	1.25 ± 0.16	0.99	1.63 ± 0.38	0.33 ± 0.02	0.99
		4	39.98 ± 1.21	0.63± 0.06	0.99	2.02 ± 1.2	0.48 ± 0.05	0.99
		8	60.97 ± 0.82	0.78 ± 0.03	0.99	5.34 ± 1.74	0.73 ± 0.1	0.98
GNR/ZIF8 50 pM	5	7.5	25.86 ± 2.51	0.35 ± 0.07	0.99	0.67 ± 0.67	0.48 ± 0.05	0.99
		10	36.06 ± 2.04	0.54 ± 0.08	0.99	1.55 ± 0.75	0.51 ± 0.04	0.99
		12.5	56.51 ± 5.77	0.44 ± 0.1	0.98	2.45 ± 0.78	0.46 ± 0.03	0.99

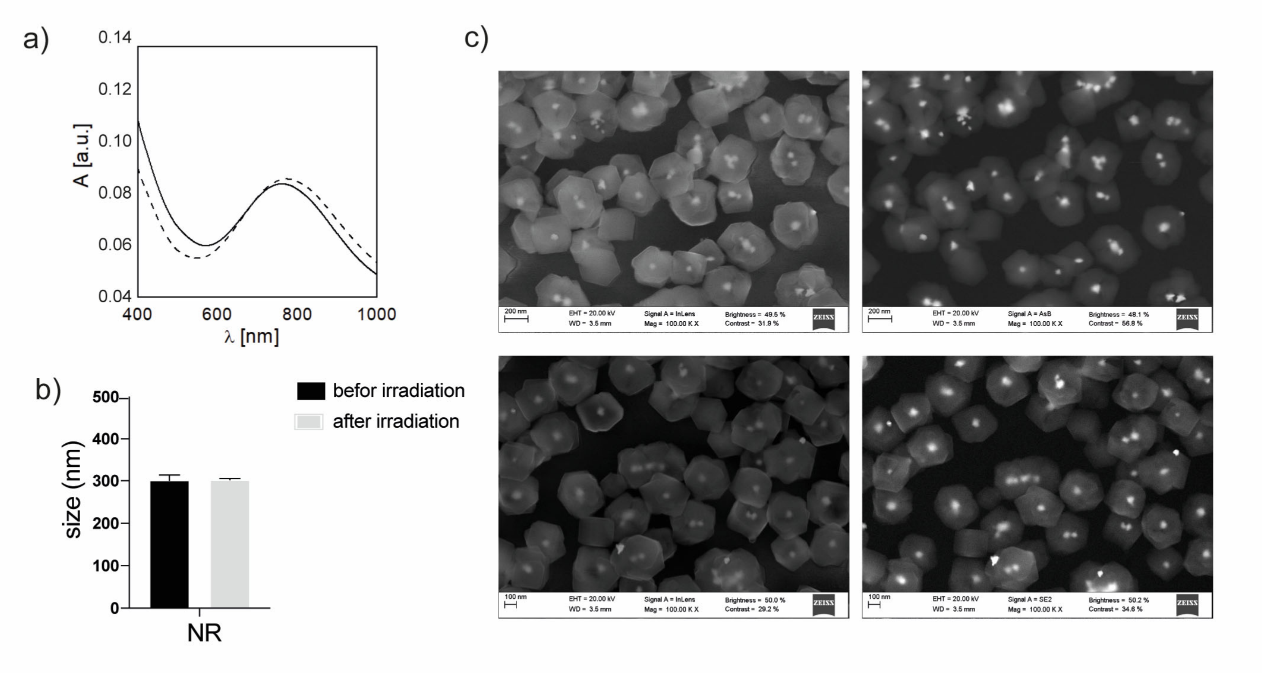

Figure S12. a) UV/Vis absorption spectra of NRs (NS2/ZFI-8) before and after laser irradiation; b) Hydrodynamic diameter d_h (mean value ± SD) given as number distributions from DLS measurements of the NRs before and after laser irradiation. SD values correspond to the standard deviation of the diameter mean value as obtained from several repetitions (n=3) of the measurements. c) SEM images of the NRs after irradiation with 808 nm laser at 8 W·cm^{-2} for 5 min. Scale bars correspond to 200 nm.

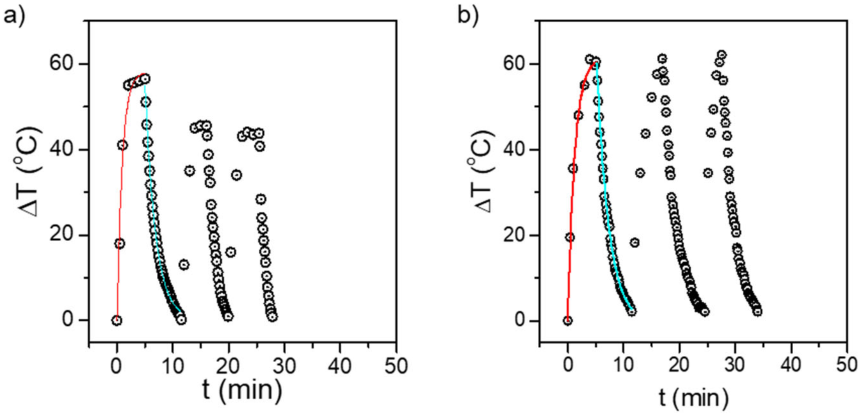

Figure S13. Reproducibility of the photothermal conversion in three successive cycles of: a) CTAB-coated NSs; b) FBS-stabilized NSs. Note that aggregation of the CTAB-coated NSs was observed after the first irradiation cycle, whereas FBS-stabilized NSs did not present any observable aggregation. The concentration of NSs particles was 0.2 nM. Irradiation conditions: 5 min at 8 W·cm^{-2}; solutions were left to cool down to RT (~22 ºC), before starting a new 5 min irradiation cycle. Solid lines represent simulation curves for heating (Box-Lucas function) and cooling (exponential) using the fitting parameters shown in Table S3.

Table S3. Fitting parameters for heating (Box-Lucas function) and cooling (exponential) simulation curves in Figure S6 and Figure 2b.

**sample**	**fitting curves**					
	**heating / $\Delta T = T_{max} \cdot (1-e^{-b \cdot t})$**			**cooling / $\Delta T = T_0 + A \cdot e^{-b \cdot t}$**		
	**T_{max} (ºC)**	**b (min^{-1})**	***R^2***	**T_0 (ºC)**	**b (min^{-1})**	***R^2***
**CTAB-coated NS**	58.08 ± 2.36	1.07 ± 0.16	0.98	0.30 ± 0.30	0.53 + 0.01	0.99
**FBS-stabilized NS**	61.44 ± 0.91	0.80± 0.04	0.99	0.00 ± 0.60	0.45 + 0.01	0.99
**NR**	55.58 ± 1.89	0.60 ± 0.05	0.99	1.21 + 0.09	0.62 ± 0.01	0.99

Performance of NR particles as nanocontainer *versus* nanoreactor

Thanks to the high porosity of the MOFs, one of their outstanding property is their ability for encapsulating large amounts of molecules inside their pores. In this way, the here synthesized NRs can act as nanocontainers for a substrate (*i.e.* probe molecule). Therefore, if on the one hand the NRs can promote a reaction, and on the other hand they can simultaneously host and carry such specific substrate, this dual role of the NRs opens the path for novel solutions in complex scenarios. Looking for example at the field of performing intracellular reactions, this could overcome problems of instability of the substrate in biological media or poor cellular uptake when the substrate is in a free form. The behaviour of our system for promoting a thermal reaction when the substrate is previously loaded inside the NRs (*i.e.* nanocontainer or loaded/filled nanoreactor) or when the NRs act simply as nanoreactor of substrate molecules diffusing freely in the surrounding environment was compared.

Loading of substrate (probe molecules) inside NRs: NRs were loaded with probe **1** before the post-functionalization with the PMA polymer. To this end, NS/ZIF-8 particles dispersed in methanol (0.5 mL, 2 nM) were mixed with a solution of the substrate **1** in methanol (150 µL, 1 mM), and the mixture was incubated overnight at RT to ensure the maximum loading, regardless of the diffusion kinetic of the molecules through the particles' pores. Then the excess of the probe **1** was removed by centrifugation, and the particles were washed once with methanol in order to remove the molecules weakly adsorbed onto the surface of the NS/ZIF-8 particles. After this, the NS/ZIF-8 particles loaded with the probe were functionalized with the PMA polymer by following the procedure described above.

***Quantification of probe loaded by fluorescence**:* To quantify the amount of the substrate **1** loaded in the NRs, the reaction was forced to achieve complete conversion of the substrate **1** into its fluorescence product **2**. To this end a solution of the substrate **1** was heated in a thermostatic bath at 90 °C, and the amount of product formed was monitored by fluorescence. After 4 h, the fluorescence intensity did not further increase, which could be attributed to the complete conversion of the substrate, as previously demonstrated by Brzezinska *et al.*[1] Then, the amount of initial substrate loaded per NR was determined by interpolation in a previously obtained calibration curve of the fluorescent product (*cf.,* Figure S14a), resulting in $1.33\times10^5 \pm 4.2 \times10^2$ molecules of probe per NR. In the case of probe **3**, quantification of **4** was performed using a calibration curve of the coumarin derivative (7-Amino-4-(trifluoromethyl)coumarin, Figure S14b).

Quantification of substrates generated: To quantify the amount of the substrates **1** and **3** transformed intro **2** and **4** by NRs after laser irradiation, a calibration curve made of a substrate **1** or **3** solution heated previously in a thermostatic bath at 90 °C was used (*cf.,* Figure S15).

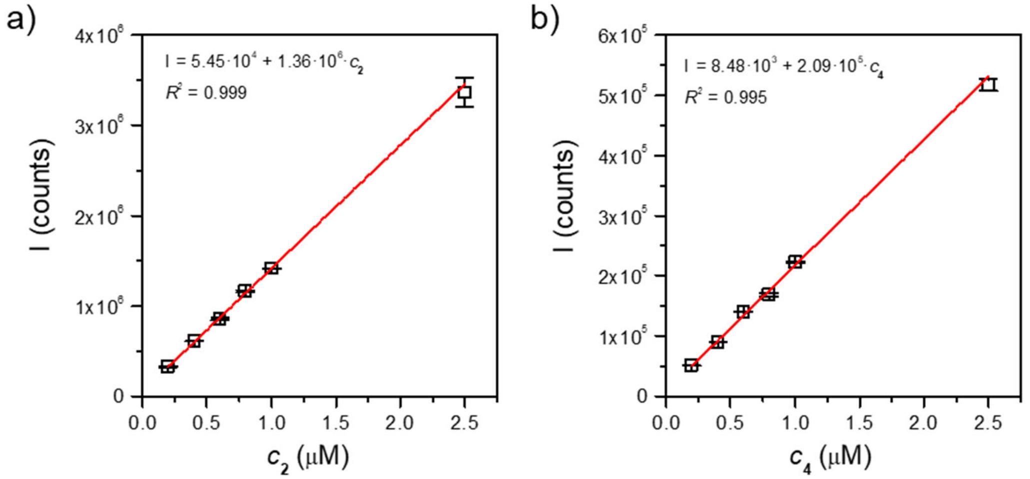

Figure S14. a) Calibration curve by using fluorescence spectrophotometer Fluorolog-3 of the fluorescent product (**2**) from the substrate **1** as obtained from fluorescence measurements under excitation (Ex.) at 350 nm. Fluorescence intensity (I) at the maximum emission (Em.) peak (λ=412 nm) as a function of the concentration is plotted, and the calibration equation is obtained by fitting a linear regression line to the collected data. b) Calibration curve of the fluorescent product (**4**) from the substrate **3** as obtained from fluorescence measurements under Ex. at 380 nm. Fluorescence intensity (I) at the maximum Em. peak (λ=490 nm) as a function of the concentration is plotted, and calibration equation is obtained by fitting a linear regression line to the collected data.

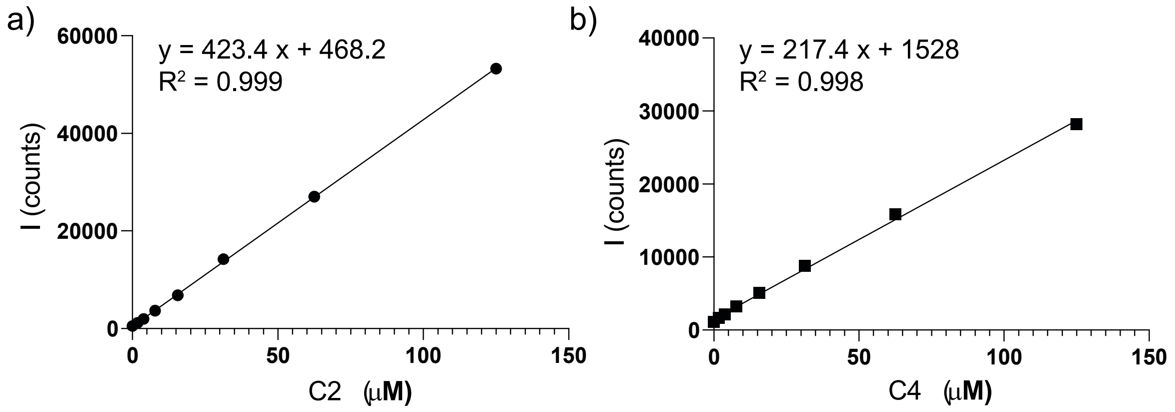

Figure S15. a) Calibration curve by using microplate reader Infinite® 200 PRO (Tecan) of the fluorescent product (**2**) from the substrate **1** as obtained from fluorescence measurements under excitation (Ex.) at 350 nm. Fluorescence intensity (I) at the maximum emission (Em.) peak (λ=412 nm) as a function of the concentration is plotted, and the calibration equation is obtained by fitting a linear regression line to the collected data. b) Calibration curve of the fluorescent product (**4**) from the substrate **3** as obtained from fluorescence measurements under Ex. at 380 nm. Fluorescence intensity (I) at the maximum Em. peak (λ=490 nm) as a function of the concentration is plotted, and calibration equation is obtained by fitting a linear regression line to the collected data.

Thermal-promoted nucleophilic substitutions by NRs

The thermoplasmonic properties of the NRs were used to promote the thermal reaction (nucleophilic substitution) depicted in Scheme S1, leading to the transformation the non-fluorescent substrates (**1** and **3**) into the corresponding fluorescent products (**2** and **4**, respectively). An aqueous solution of NRs (200 µL, 0.2 nM) was mixed with the solution of the substrate (various concentrations) in a well of a 96-well plate, and the mixture was irradiated with our 808 nm NIR set up. Different power densities (2, 4, and 8 $W \cdot cm^{-2}$) and irradiation times (from 0 to 10 min) were studied. After irradiation, the temperature of the solution was measured (T_{max}), the NRs were then precipitated by centrifugation, and the generated product in the supernatant was quantified by fluorescence using the corresponding calibration curve (Figure S15). Data are presented in Figure 3d, showing that experiments involving varying NR-to-substrate molar ratios (mol% NR) achieve the maximum transformation of **1** into **2,** and **3** into **4.**
To compare the effect of irradiation with simply bulk heating using a bath, control experiments by heating the reaction mixture in a thermostatic bath at 80 °C during 5 min were carried out, leading to negligible yields (Figure S16 and Table S4). Additionally, manifold controls (*i.e.*, the performance of ZIF-8 particles without NS core, FBS-stabilized NS2, and substrate without photocatalyst) were carried out, as summarized in Table S4.

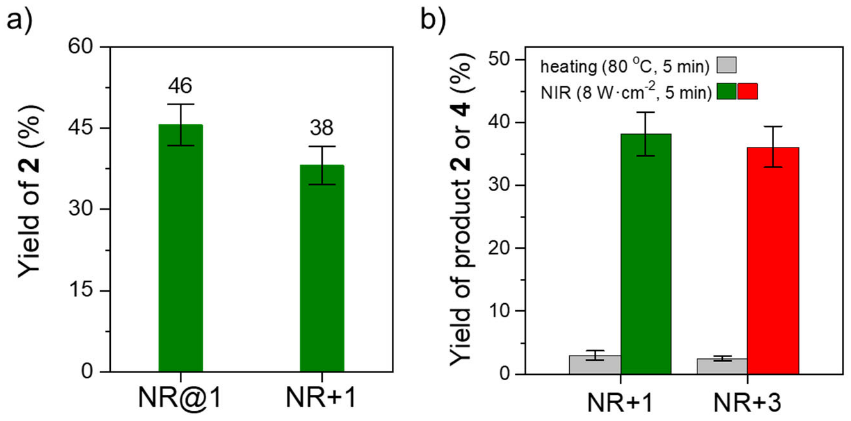

Figure S16. a) Yield of **2** after NIR treatment of a water suspension of substrate 1 (8 $W \cdot cm^{-2}$, 5 min); NRs dispersed in water with substrate **1**, either encapsulated in the NR (NR@**1**) or "free" in solution (NR+**1)**, ratio NR:**1** = $7.7 \cdot 10^{-4}$ mol% NR. b) Comparison of yield of **2** or **4** after NIR (8 $W \cdot cm^{-2}$, 5 min); NRs dispersed in water (0.2 nM) with either **1** or **3**, respectively.

Table S4. Control experiments for reaction yields of substrates (**1** and **3**) to generate the fluorescent products (**2** and **4**) promoted by NRs (FBS-stabilized NS and without catalyst included) under different conditions to study the following: i) Laser irradiation conditions (irradiance, irradiation time), and ii) thermoplasmonic heating *versus* conventional heating in a water bath.

**Particle (0.2 pmol)**	**NIR Irradiance ($W \cdot cm^{-2}$)**	**Time (min)**	**T_{max} (ºC)**	**T_{bath} (ºC)**	**Substrate (100 nmol)**	**Product / Yield (%) ± SD****
NR	2	2	32	none	**1**	n.r.*
NR	2	5	45	none	**1**	n.r.*
NR	2	10	60	none	**1**	**2** / 0.5 ± 0.3
NR	4	2	43	none	**1**	**2** / 0.3 ± 0.1
NR	4	5	57	none	**1**	**2** / 1.9 ± 0.1
NR	4	10	75	none	**1**	**2** / 3.5 ± 0.3
none	8	5	22	none	**1**	**2** / 2.9 ± 0.7
none	none	5	80	80	**1**	n.r.*
"bare" ZIF-8#	none	5	80	80	**1**	n.r.*
"bare" ZIF-8#	8	5	22	none	**1**	**2** / 2.0 ± 0.8
NS/FBS$	8	5	81	none	**1**	**2** / 5.0 ± 0.5
NR	none	5	80	80	**1**	**2** / 2.9 ± 0.7
NR	none	5	80	80	**3**	**4** / 2.5 ± 0.4

*n.r.: no reaction; # "bare" ZIF-8 nanoparticles equivalent to NRs, but without the NS core; $NS/FBS correspond to FBS-stabilized NSs; **SD values correspond to the standard deviation as obtained from several repetitions (n=3) of the measurement.

Photostability and reusability of the NRs

The photostability of the NRs after irradiation is a critical point for their potential reuse in successive cycles, and thus it was investigated. As previously shown, SEM images of the NR particles after irradiation were acquired, which showed that there were no changes on their morphology and size, *cf.*, Figure 3c and Figure S7. Second, the performance of the NRs for promoting the thermal reaction in five successive steps was studied as follows: for 1 cycle, a solution of NRs was mixed with the solution of the substrate (NR:1 = $1\cdot10^{-3}$ mol% NR) in a well of a 96-well plate, and the mixture was irradiated with a 808 nm laser at 8 $W\cdot cm^{-2}$ for 5 min; then the NRs were precipitated by centrifugation and the generated product in the supernatant was quantified by fluorescence. For 2 successive cycles, an identical mixture of NRs with substrate placed in a well was irradiated using 8 $W\cdot cm^{-2}$ for 5 min. The mixture was left to cool down until initial temperature conditions (*i.e.* waiting 10 min for assuring that RT was reached). Then, the irradiation was repeated for another 5 min, and after cooling the NRs were precipitated by centrifugation and the generated product in the supernatant was measured by fluorescence. The same process was repeated up to a total of 3 cycles. Yields are presented in Figure S16a and Table S5, which indicates that NRs can act as a truly nanoreactor. Equivalent experiments using 8 $W\cdot cm^{-2}$ for 2 min, 5 consecutive irradiation/cooling cycles are presented in Figure S16b and Table S5.

Finally, the reusability was also tested by performing the reaction using the same NRs mixed with the substrate **1** (NR:1 = $1\cdot10^{-3}$ mol% NR) in five repetitive cycles at 8 $W\cdot cm^{-2}$ for 5 min. To this end after each irradiation cycle, *i.e.,* after each use, the NRs were isolated by centrifugation and redispersed in a fresh aqueous solution containing the substrate for a new irradiation cycle (*i.e.,* run). The supernatants from each use were measured by fluorescence to quantify the amount of generated product in each run. Data presented in Figure S16c and Table S5, clearly shows that negligible changes were observed after five reuses.

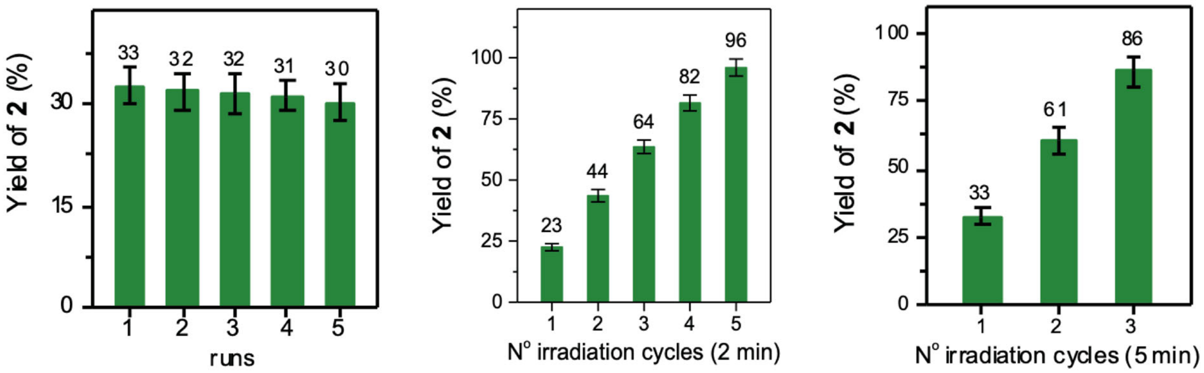

Figure S17. a) Reusability of NRs; yield of **2** after different NIR cycles (8 $W\cdot cm^{-2}$, 5 min NIR and washing); NRs dispersed in water with substrate **1**; ratio NR:**1** = $1\cdot10^{-3}$ mol% NR. b) Cumulative yield of **2** after different NIR cycles (8 $W\cdot cm^{-2}$, 2 min NIR and 5 min cooling to room temperature); NRs dispersed in water with substrate **1**; ratio NR:**1** = $1\cdot10^{-3}$ mol% NR. c) Cumulative yield of **2** after increasing number of NIR cycles (8 $W\cdot cm^{-2}$, 5 min NIR and 5 min cooling to room temperature); NRs dispersed in water with substrate **1**; ratio NR:**1** = $1\cdot10^{-3}$ mol% NR.

Table S5. Thermal reaction yields of substrate **1** to generate the fluorescent product **2** promoted by NRs to study the following: i) the performance of the NRs as reactor by performing five successive irradiation cycles (8 W·cm^{-2}, 2 min each one, 1·10^{-3} NR%) and without the isolation of the NRs nor the reactants between one irradiation and the following one. Cumulative yields (%) are presented; ii) equivalent to (i) but with three successive irradiation cycles (8 W·cm^{-2}, 5 min each one, 100 nmol of **1**); iii) the reusability of the NRs by performing five repetitive reuses, involving the isolation of the NRs after each use and redispersion in a fresh substrate (**1,** 1·10^{-1} NR%) solution for a new irradiation (8 W·cm^{-2}, 5 min).

**(i) Irradiation cycles**		**(ii) Irradiation cycles**		**(iii) Runs**	
**Nº cycles**	**Yield (%)***	**Nº cycles**	**Yield (%)***	**Run No.**	**Yield (%)**
1	22.5 ± 1.4	1	32.7 ± 2.7	1	32.7 ± 2.7
2	43.6 ± 2.5	2	61.1 ± 4.9	2	31.9 ± 2.8
3	63.6 ± 2.8	3	86.3 ± 5.6	3	31.6 ± 2.9
4	81.5 ± 3.2	--	--	4	31.2 ± 2.3
5	96.0 ± 3.5	--	--	5	30.3 ± 2.8

*Cumulative yield

Cell studies

Cell culture. HeLa (cervical cancer cell line) were cultured in Dulbecco's Modified Eagle Medium with phenol red, 4.5 g/L D-glucose, L-glutamine and pyruvate (DMEM, 1X, Gibco, #41966-029) supplemented with 10% Fetal Bovine Serum (Gibco, #10270-106) and 1% Penicillin Streptomycin (P/S, Corning, 100X, #30-002-CI). Cells were maintained under humid conditions at 37 °C and 5% of CO_2. Cells were passaged with 0.25% Trypsin-EDTA (1X, Gibco, 25200-056) when the culture reached confluency, after cleaning (Dulbecco's Phosphate Buffered Saline, DPBS, 1X, Gibco, #14190-094).

Cell Viability. In order to study number of viable cells after the exposure to the substrates or particles (NS or NR), resazurin assays were performed (Figure S18). HeLa were seeded in 96-well plates (NEST Scientific, #701001), $7.5 \cdot 10^3$cells per well in 100 µL of cell growth medium (0.3 cm^2 *per* well) 24 h before treatments. Then media was removed and 100 µL of cell culture growth medium with the desired concentration of the substrates or the particles were added. The cells were incubated with the samples the time of interest at 37 °C and 5% CO_2. Then, each well was rinsed three times with PBS and 100 µL of a freshly prepared solution with 90% of media and 10% of resazurin (resazurin sodium salt in water 0.2 mg/mL filtered; Resazurin Sodium Salt, Sigma Aldrich, #199303-1G) was added. Cells were incubated at 37 °C and 5% CO_2 under dark conditions. After irradiations, cells were maintained overnight before adding resazurin solution.

Non-fluorescent resazurin (alamarBlue™) is oxidized by living cells into its fluorescent product resorufin (Ex. 579 nm / Em. 584 nm). In this way, the fluorescence intensity of each well is proportional to the number of living cells there. After the incubation time (4 hours), plates were measured with a plate reader (Infinite® 200 PRO, Tecan, Switzerland) under Ex./Em. wavelengths 560(20)/610(20) nm. The fluorescence value of each well provided by the instrument is an average of nine consecutive measures in the same well. The final intensity value for control cells (I_C), the ones that were not treated, is an average of, at least, nine different well values. The final intensity values for samples (I_S) are a mean of three independent well values. So, we can calculate the final cell viability values as:

$$\text{cell viability (\%)} = \frac{I_S}{I_C} \cdot 100$$

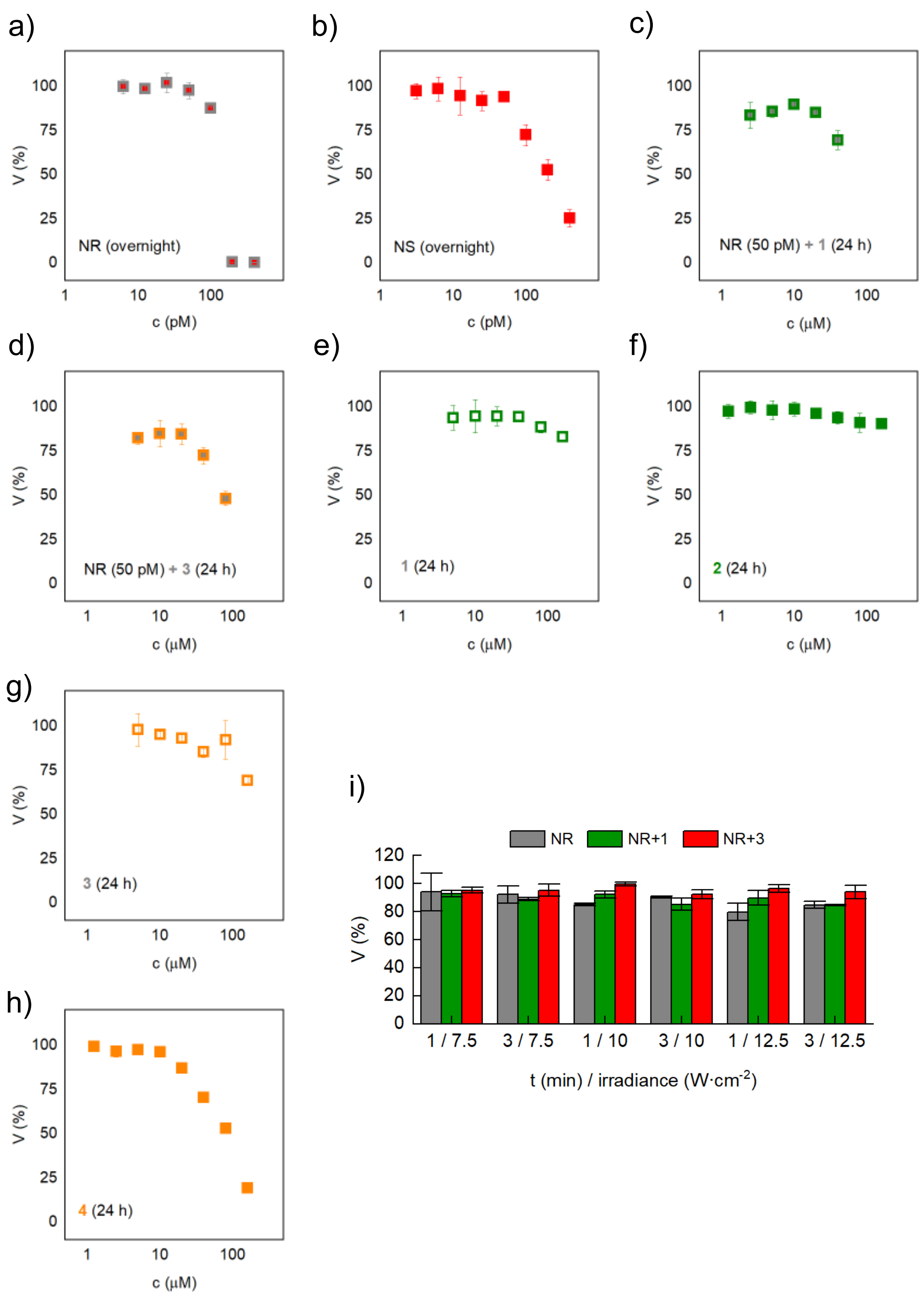

Figure S18. Cell viability using the resazurin assay of HeLa cells exposed to increasing concentrations of reactant(s) and/or NRs and/or FBS-stabilized NSs. a) NRs (50 pM), 24 h incubation; b) NSs (50 pM), 24 h incubation; c) **1**, 24 h incubation with NR (50 pM)-preloaded cells; d) **3**, 24 h incubation with NR (50 pM)-preloaded cells; e) **1**, 24 h incubation; f) **2**, 24 h incubation; g) **3**, 24 h incubation; h) **4**, 24 h incubation; i) NIR treatments, varying exposure time t (1 or 3 min) and irradiance (7.5, 10 and 12.5 W·cm^{-2}), of NR (50 pM)-preloaded cells, either with 24 h incubation with **1** (10 µM, green bars) and 3 (10 µM, red bars) or in the absence of substrate (grey bars). Data expressed as mean ± SD, n=3.

Au content per cell. ICP-MS was used to quantify the average gold content per cell, which we used to estimate the number of FBS-stabilized NSs or NRs per cell. HeLa cells (~ $5\cdot10^6$ cell per experiment) were cultured as previously discussed, supplemented with FBS-stabilized NSs or NRs (50 pM), and incubated overnight. Extracellular gold (either free or particles) was rinsed with PBS (3x), and the particle-loaded cells were digested with aqua regia after counting them. Digested samples were diluted with HCl 2% before quantifying gold content by ICP-MS. For comparison of particle uptake (NR or NS) among samples, we assumed that the gold mass of one NS is ~ $8\cdot10^{-16}$ g (*i.e.*, $4.1\cdot10^4$ nm^3),[5] which allowed us to make an estimation about the average particle uptake per cell: (335 ± 12) NRs/cell and (166 ± 61) NSs/cell.

Cytometry. Internalization of NRs over time was qualitatively monitored by cytometry studies using TAMRA-labeled NRs (see section S2). $15\cdot10^3$ HeLa cells (300 µL) were seeded on 48 well plates and let 24 hours to attach. After the NRs application in complete DMEM during different times (50 pM, 0.5 - 24 h), cells were washed three times with PBS to remove non-internalized NRs. Then, 50 µL of trypsin were added to each well. After 5 minutes, 150 µL of PBS was added before measuring cells in the cytometer. Mean fluorescence intensity (MFI) was analyzed to compare the amount of NRs internalized by cells at different times after incubation (Figure S19).

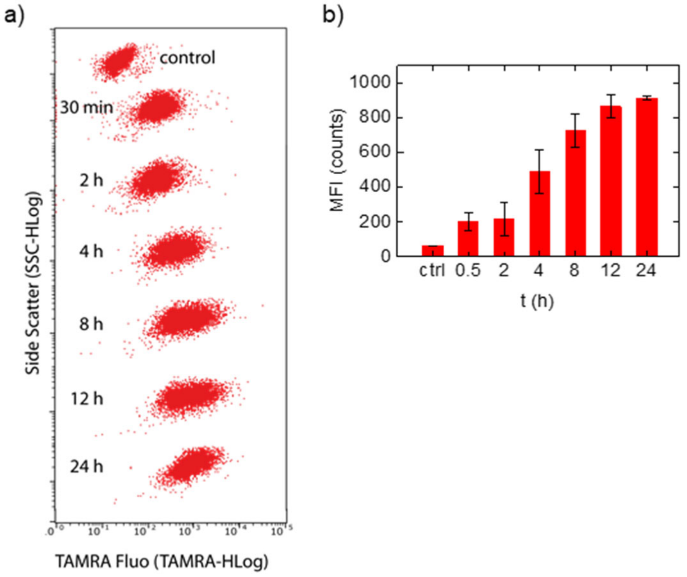

Figure S19. a) Scatter density plots of SSC signal *versus* fluorescence signal (channel Orange-G, Ex. 532 nm, Em. 620/52 nm) for TAMRA-labeled NRs; b). Quantification of the MFI of the cellular uptake of TAMRA-labeled NRs in HeLa cells after different incubation times. Data expressed as mean ± SD, n=3.

Laser irradiations. After checking that the NR-preloaded cells with the added substrate (**1** or **3**, 10 µM, 30 min incubation) appear morphologically normal under the microscope, we chose one of the following irradiation protocols: i) highly focused NIR spot (high power densities of a 785 nm laser "pointer" with diameter ~ 5 µm, as in classical optical tweezers) for single cell experiments; ii) illumination of thousands of cells with a large diameter (~ 0.65 cm; low power density) collimated NIR beam, for which we used a 808 nm laser (see section IV, Figure S10). Since the beam is collimated, we consider a homogeneous spot and thus, we can obtain the intensity just dividing the power by the surface (in cm^2) of the spot. We explored different irradiation conditions (exposure time and irradiance). For high area irradiation aimed to achieve intracellular conversion of substrates (**1** or **3**, 10 µM, 30 min incubation) into products (**2** and **4**), we used 10 $W\cdot cm^{-2}$, 1 min. For single cell experiments using the NIR pointer, we used exposure times ~5-10 s.

Confocal imaging: In order to perform all the confocal imaging experiments with living cells, $20 \cdot 10^3$ HeLa cells (200 µL) were seeded on µ-Slide 8 well-ibiTreat chambers (1 cm^2 *per* well, Ibidi, Germany, #80826) at least 12 h before particle/substrate incubation. Confocal images of living cells were acquired with different magnification objectives (20x, 60x, 100x). All the images were processed with ImageJ (Figures S20-S27).

The following Ex./Em. wavelengths were used for confocal imaging:

- 561/620(60) for TAMRA-labeled NRs (Figure S20);
- 637/725(40) nm for cell plasma membrane staining using CellMask™ Deep Red (Figure S20);
- 405/450(50) nm or 488/525(50) nm, respectively, for product **2** after NIR excitation using a NIR focused pointer (*i.e.* optical setup equivalent to optical tweezers,[6] Figure S21) or a homogeneous circular beam (diameter ~ 0.65 cm, as previously described in Figure S10; see Figure S24);
- 405/525(50) nm or 540/620(60) nm for product **4** after NIR excitation using the focused pointer (Figures S22 and S23) or the circular beam (Figures S26 and S27), respectively.

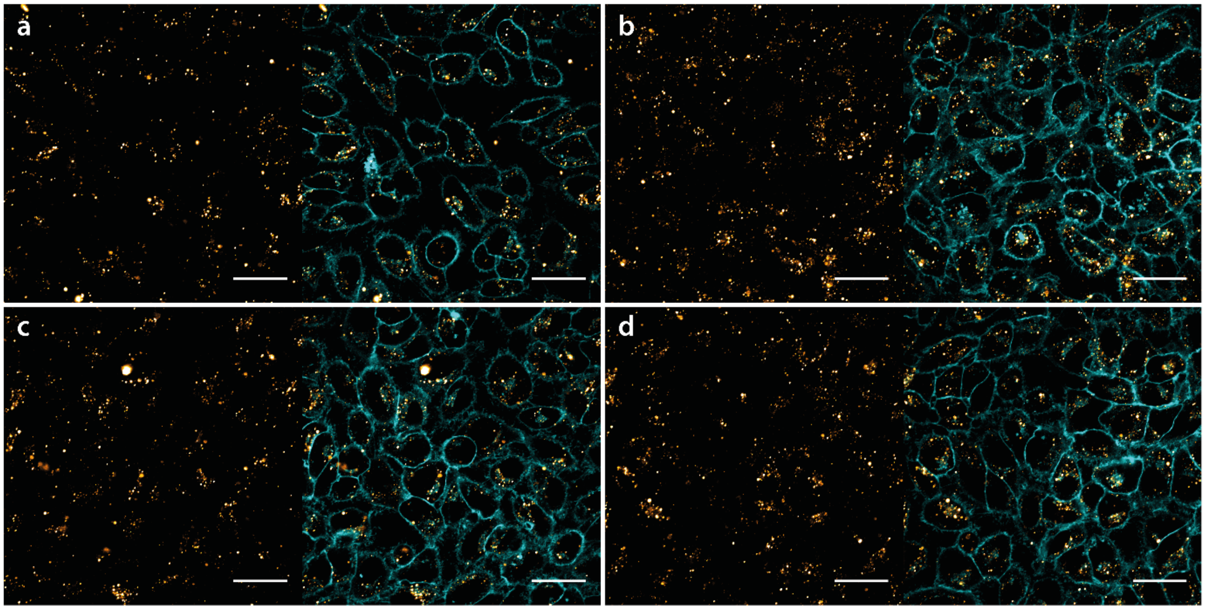

Figure S20. a-d) Confocal microscopy images: TAMRA-labeled NRs (orange; Ex./Em.: 561/620(60) nm); TAMRA-labeled NRs (orange) + cell plasma membrane staining using CellMask™ Deep Red (cyan; Ex./Em.: 637/725(40) nm). Scale bars correspond to 40 µm.

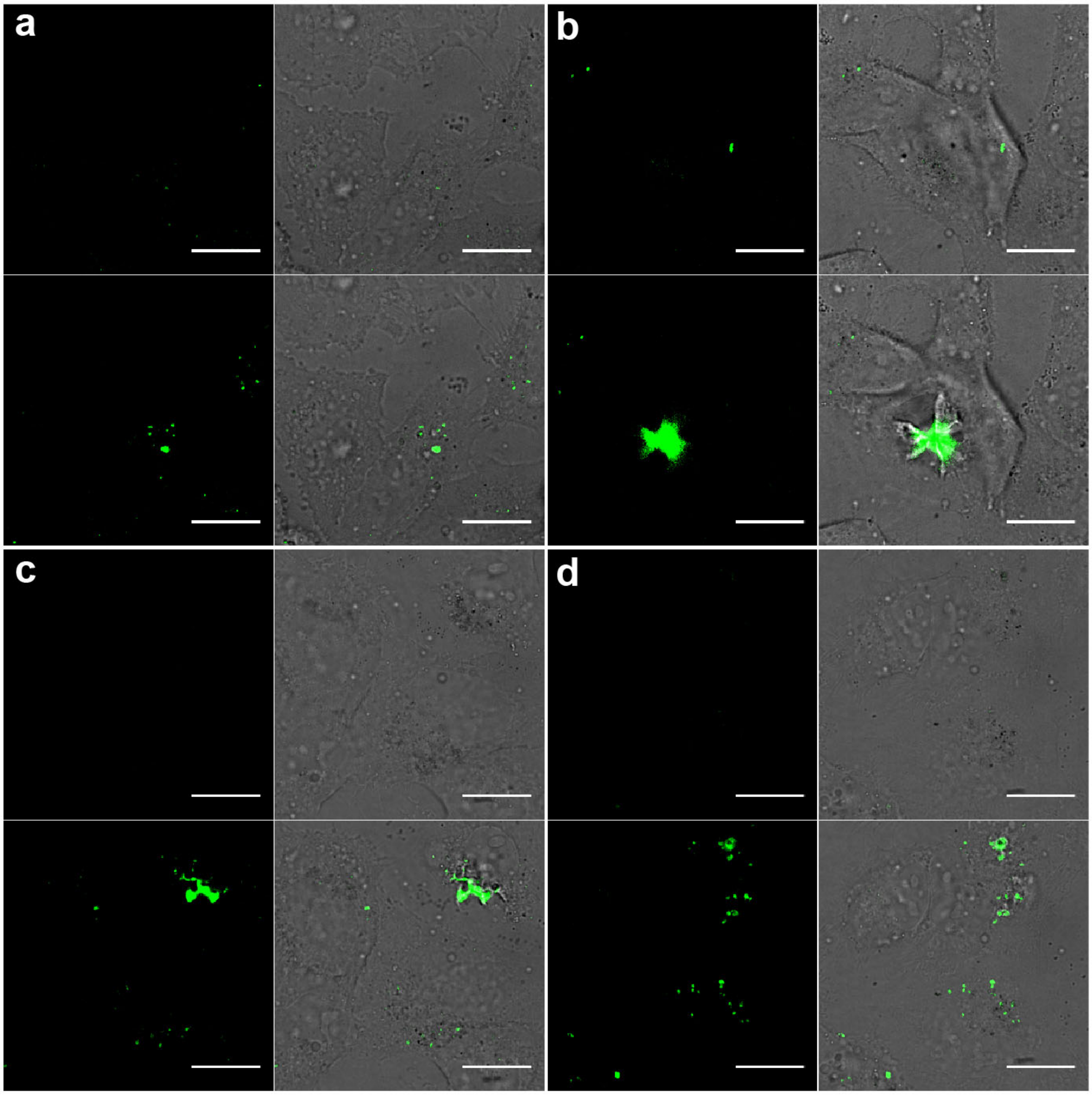

Figure S21. a-d) Confocal microscopy images of NR@**1**-preloaded cells, before (top) and after (down) NIR pointer irradiation; fluorescence (left, Ex./Em. = 405/450(50) nm) and merged (right) bright field + fluorescence images are shown. Scale bars correspond to 20 µm.

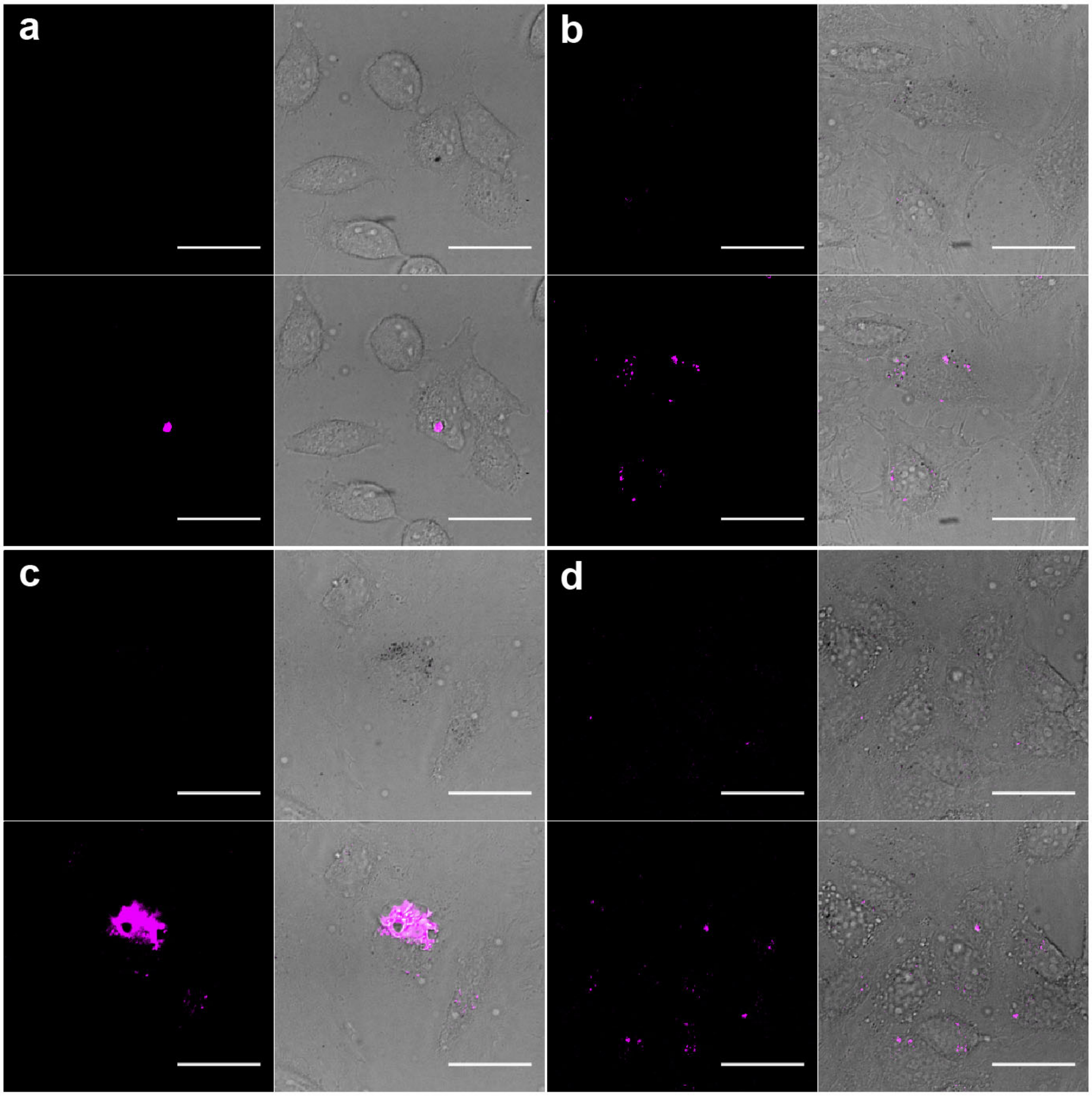

Figure S22. a-d) Confocal microscopy images of NR@**3**-preloaded cells, before (top) and after (down) NIR pointer irradiation; fluorescence (left, Ex./Em. = 405/525(50) nm) and merged (right) bright field + fluorescence images are shown. Scale bars correspond to 20 µm.

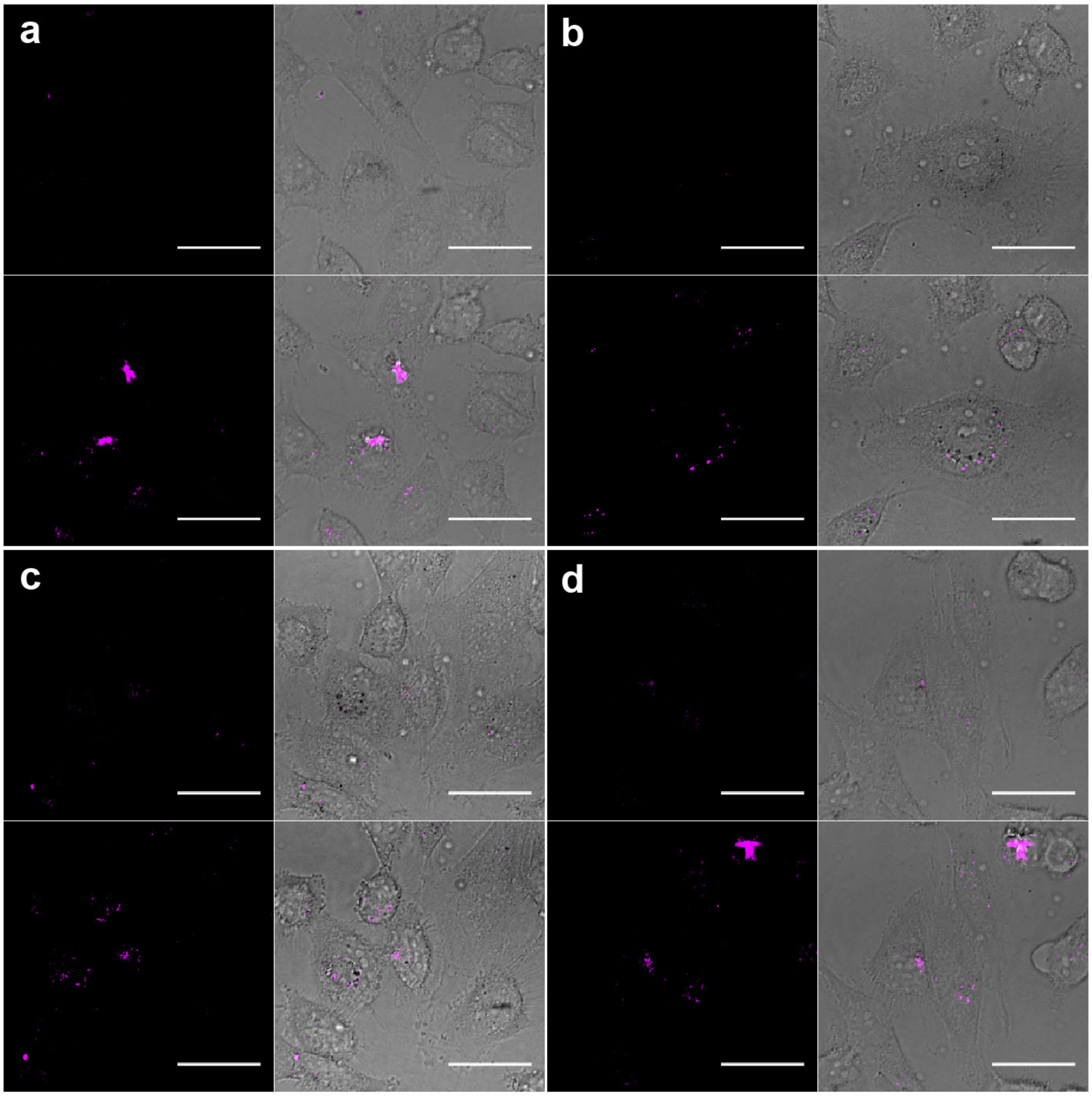

Figure S23. Confocal microscopy images of NR-preloaded cells incubated with **3** (10 μM, 30 min), before (top) and after (down) NIR pointer irradiation; fluorescence (left, Ex./Em. = 405/525(50) nm) and merged bright field + fluorescence images are shown. Scale bars correspond to 20 μm.

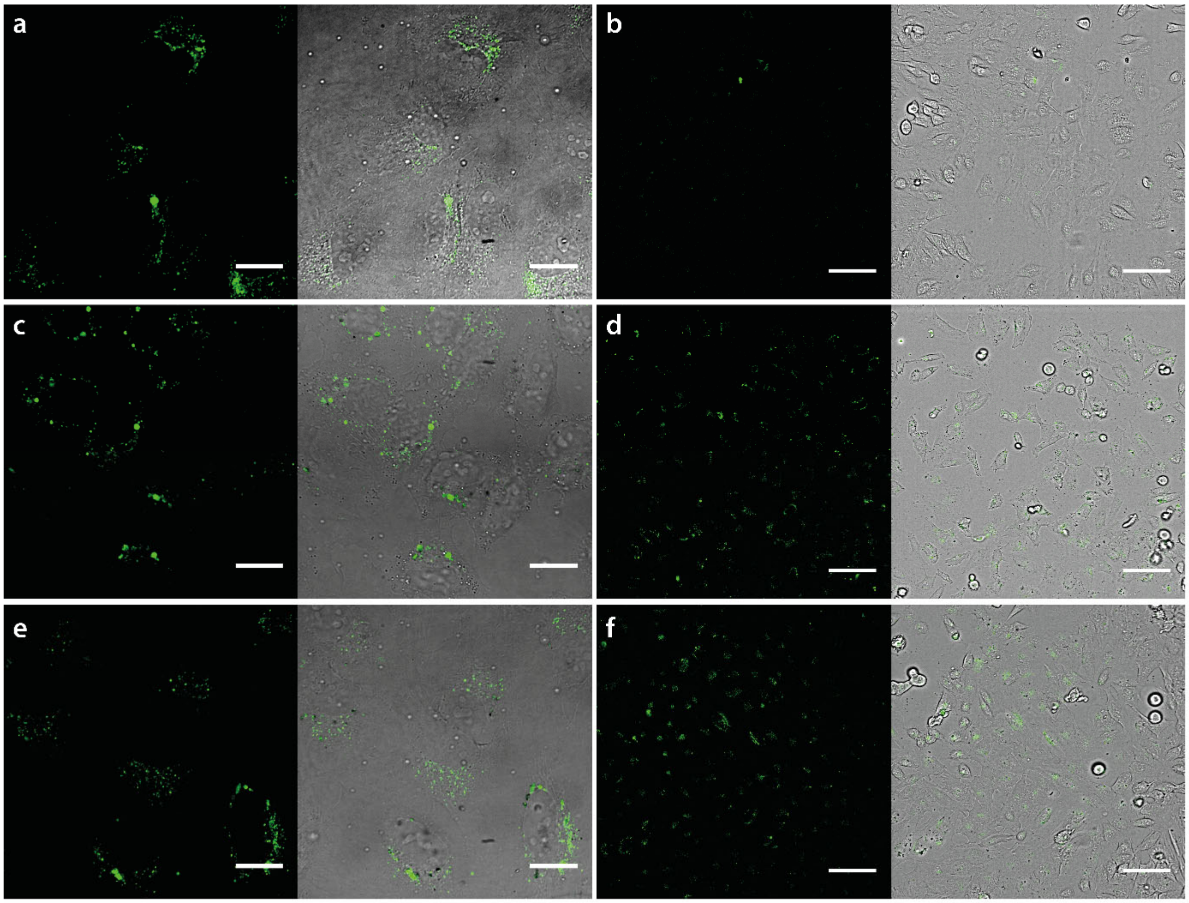

Figure S24. Controls with probes **1** or **2** (10 µM, 30 min), and without NIR. a, b) control cells without NRs, incubated with **1**; c, d) control NR-preloaded cells (50 pM, overnight) incubated with **1**; e, f) control NR-preloaded cells (50 pM, overnight) incubated with **2** (10 µM, 30 min). Fluorescence (left, Ex./Em. = 488/525(50) nm) and merged bright field + fluorescence images are shown. Scale bars are 20 µm (a, c, e; 100x) or 100 µm (b, d, f; 20x).

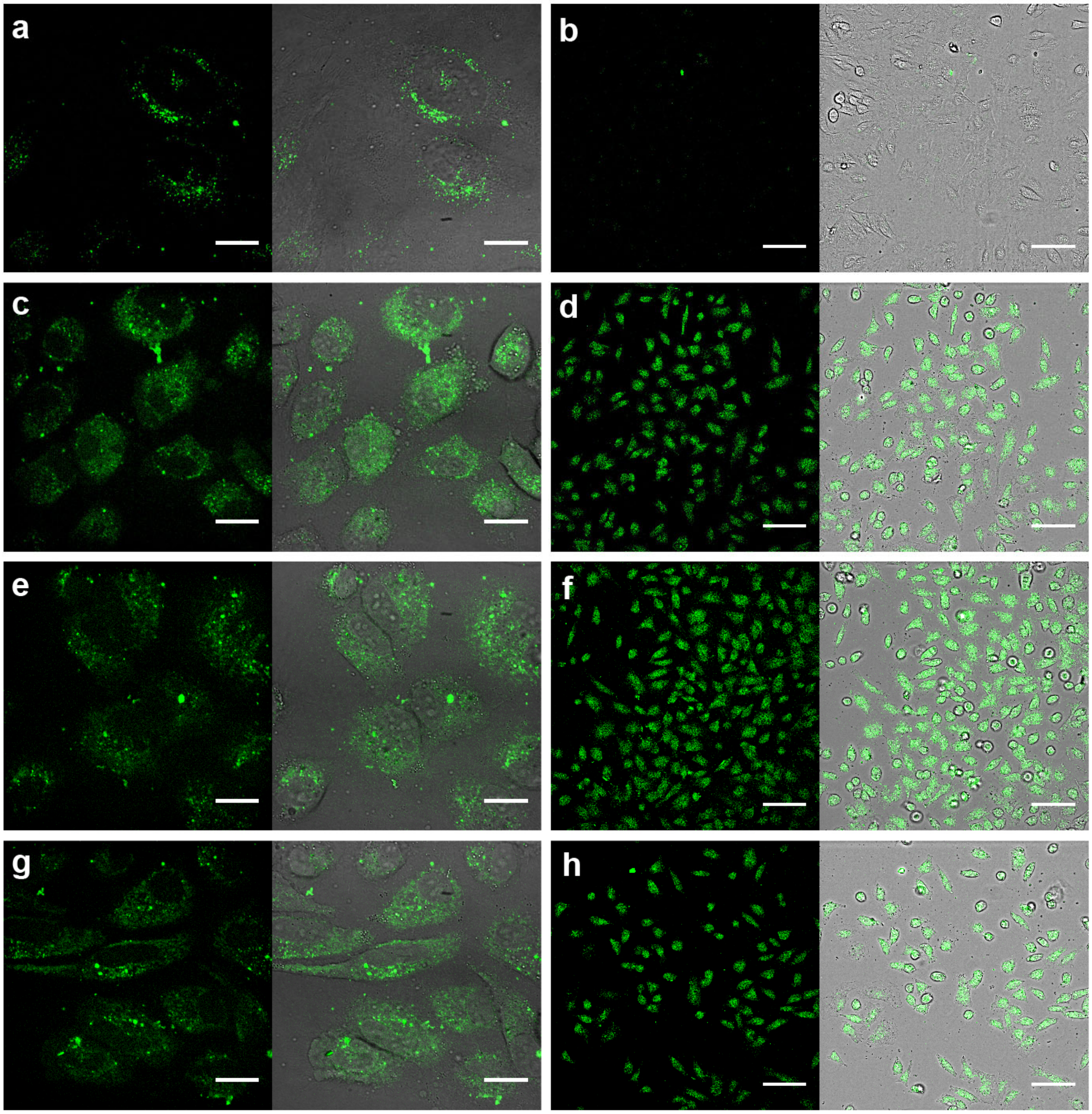

Figure S25. Particle (NS or NR, 50 pM overnight)-loaded cells with substrate **1** (10 μM, 30 min) and NIR (10 W·cm^{-2}, 1 min). a, b) NS-preloaded cells; c-h) NR-preloaded cells. Fluorescence (left, Ex./Em. = 488/525(50) nm) and merged bright field + fluorescence images are shown. Scale bars are 20 μm (a, c, e, g; 100x) or 100 μm (b, d, f, h; 20x).

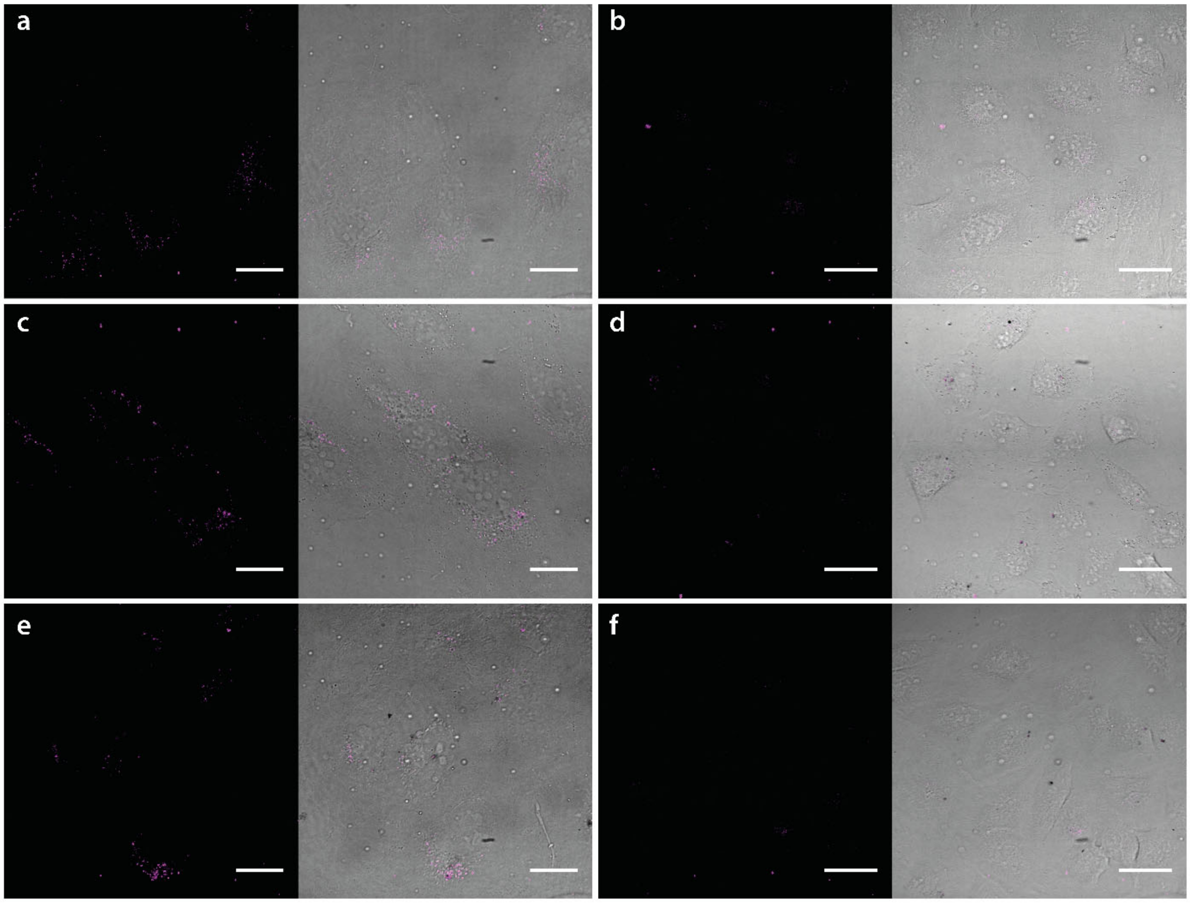

Figure S26. Controls with probes **3** or **4** (10 µM, 30 min), and without NIR. a, b) control cells without NRs, incubated with **3**; c, d) control NR-preloaded cells (50 pM, overnight) incubated with **3**; e, f) control NR-preloaded cells (50 pM, overnight) incubated with **4** (10 µM, 30 min). Fluorescence (left, Ex./Em. = 540/620(60) nm) and merged bright field + fluorescence images are shown. Scale bars are 20 µm (a, c, e; 100x) or 40 µm (b, d, f; 60x).

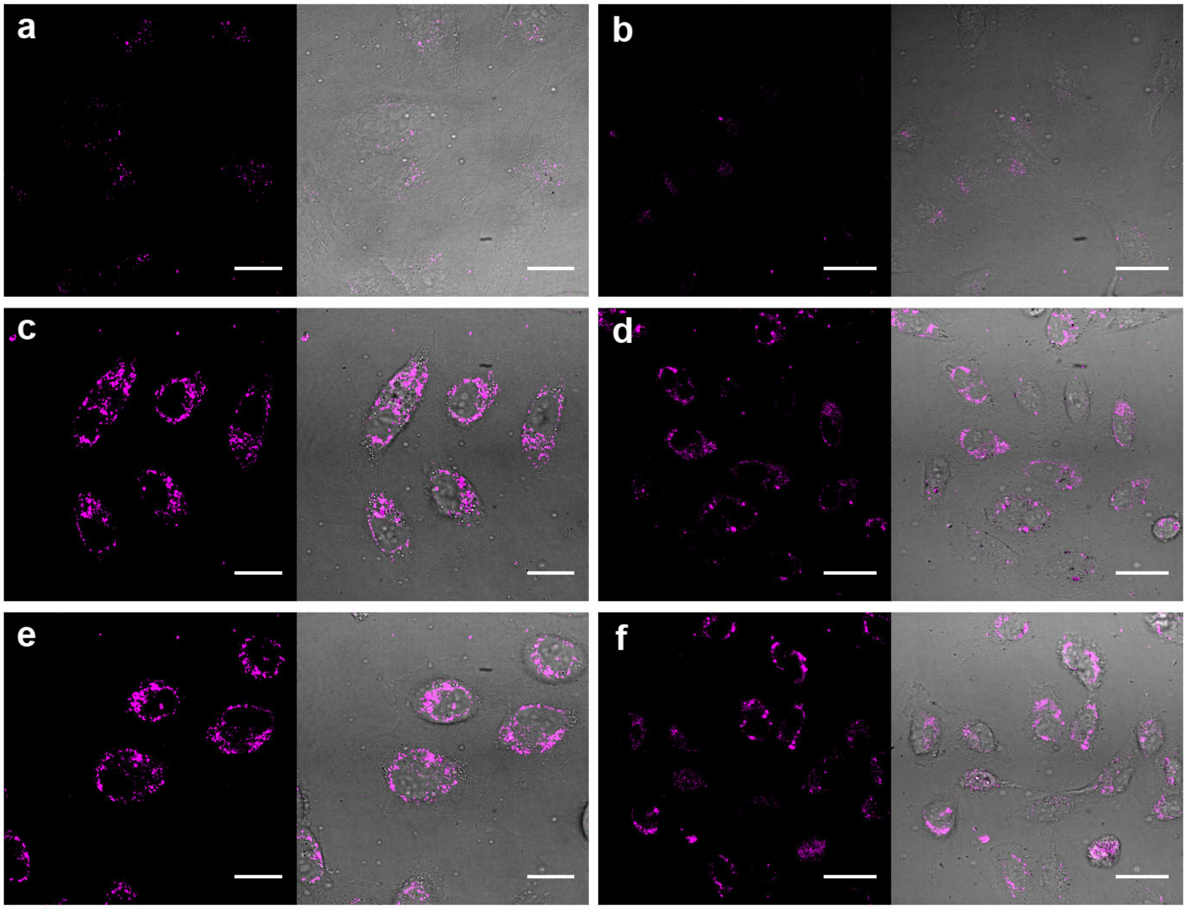

Figure S27. Particle (NS or NR, 50 pM overnight)-loaded cells with substrate **3** (10 µM, 30 min) and NIR (10 W·cm^{-2}, 1 min). a, b) NS-preloaded cells; c-f) NR-preloaded cells. Fluorescence (left, Ex./Em. = 540/620(60) nm) and merged bright field + fluorescence images are shown. Scale bars are 20 µm (a, c, e; 100x) or 40 µm (b, d, f; 60x).

References

1. Brzezinska, J.; Witkowska, A.; Bałabańska, S.; Chmielewski, M. K., 2-Pyridinyl-N-(2,4-Difluorobenzyl)Aminoethyl Group as Thermocontrolled Implement for Protection of Carboxylic Acids. *Org. Lett.* **2016,** *18*, 3230-3233.
2. Chmielewski, M. K., Novel Thermolabile Protecting Groups with Higher Stability at Ambient Temperature. *Tetrahedron Lett.* **2012,** *53*, 666-669.
3. Chmielewski, M. K.; Tykarska, E.; Markiewicz, W. T.; Rypniewski, W., Engineering N-(2-Pyridyl)Aminoethyl Alcohols as Potential Precursors of Thermolabile Protecting Groups. *New J. Chem.* **2012,** *36*, 603-612.
4. Hühn, J.; Carrillo-Carrion, C.; Soliman, M. G.; Pfeiffer, C.; Valdeperez, D.; Masood, A.; Chakraborty, I.; Zhu, L.; Gallego, M.; Yue, Z.; Carril, M.; Feliu, N.; Escudero, A.; Alkilany, A. M.; Pelaz, B.; del Pino, P.; Parak, W. J., Selected Standard Protocols for the Synthesis, Phase Transfer, and Characterization of Inorganic Colloidal Nanoparticles. *Chem. Mater.* **2017,** *29*, 399-461.
5. Tsoulos, T. V.; Han, L.; Weir, J.; Xin, H. L.; Fabris, L., A Closer Look at the Physical and Optical Properties of Gold Nanostars: An Experimental and Computational Study. *Nanoscale* **2017,** *9*, 3766-3773.
6. Kantner, K.; Rejman, J.; Kraft, K. V. L.; Soliman, M. G.; Zyuzin, M. V.; Escudero, A.; del Pino, P.; Parak, W. J., Laterally and Temporally Controlled Intracellular Staining by Light-Triggered Release of Encapsulated Fluorescent Markers. *Chem. Eur. J.* **2018,** *24* (9), 2098-2102.
